# Degradation of Exhaust Valves in Medium-Speed Marine Diesel Engines: An Evidence-Tiered Review of Materials, Surface Engineering, and Qualification

**DOI:** 10.3390/ma19194166

**Published:** 2026-09-29

**Authors:** Lihe Jiang, Shuaikang Wang, Pengyang Li, Jinwei Long, Yanhong Wei

**Affiliations:** 1 College of Materials Science and Technology, Nanjing University of Aeronautics and Astronautics, 29 Jiangjun Road, Nanjing 211106, Chinajinweilong@nuaa.edu.cn (J.L.); 2 COSCO Shipping Marine Equipment & Spares (Nanjing) Co., Ltd., 18 Tangwei Road, Nanjing 211121, China; lpy007@foxmail.com; 3 Jiangsu Engineering Technology Research Center for Advanced Manufacturing of Ship Power System Components, Nanjing 211121, China

**Keywords:** marine diesel engine, exhaust valve, thermo-mechanical fatigue, hot corrosion, valve seat wear, hardfacing, remanufacturing, evidence synthesis

## Abstract

Exhaust valves in medium-speed marine diesel engines experience coupled combustion heating, intermittent seat cooling, repeated impact and microslip, deposit-assisted corrosion, and prolonged service. Their degradation therefore cannot be explained or qualified using a single room-temperature property. This review organizes the evidence within a mechanism–material–process–performance framework and separates marine observations from transferable valve studies and general materials data. A reference-seeded narrative synthesis was supplemented by a documented literature update completed in September 2026. Quantitative comparisons retain test temperature, geometry, exposure, measurement basis, and transfer limitations. The reported 10^7^-cycle strength of 42Cr9Si2 fell from approximately 569 MPa at 400 °C to 300 MPa at 650 °C. Moreover, two marine hardfacings with overlapping post-service hardness ranges exhibited markedly different damage. Together, these findings indicate that hot strength, scale stability, contact evolution, thermal transport, dilution, interface integrity, and repair history must be assessed jointly. Deposit-assisted corrosion, contact degradation, and leakage may form reinforcing feedback, although no universal time-resolved sequence has been validated. The proposed qualification framework links material and surface engineering decisions to staged coupon, component, engine, and field evidence. It also identifies valve-specific datasets, coupled test protocols, repeated repair validation, and uncertainty-aware prognosis as priority research needs.

## 1. Introduction

Medium-speed four-stroke diesel engines operate for long periods under load transients, high cylinder pressures, and constrained overhaul windows. The exhaust valve must retain structural integrity, dimensional stability, gas tightness, and effective heat conduction through the seat and stem. Marine thermal models predict head temperatures near 700 °C, but the estimates depend on heat transfer coefficients, contact conditions, material architecture, and operating point [1,2]. They therefore do not define a universal service limit (Tier A). At an assumed 500–1000 rpm, one closure every two revolutions produces 15,000–30,000 closures h^−1^. This corresponds to 1.5–3.0 × 10^8^ seating events over 10,000 h. The author-derived estimate describes exposure frequency, not independent TMF cycles or a validated overhaul interval.

Service evidence also shows why valve failure cannot be reduced to a single material property deficit. In a 6SW280 engine, a fatigue fracture of one valve head caused extensive secondary damage to the paired valve, seats, cylinder head, piston, and turbocharger [3]. Another medium-speed marine engine study found markedly different damage after approximately 2000 and 3000 h in two cobalt-alloy hardfacings [4,5]. The finding shows that labels such as “cobalt-based” or “Stellite” do not define dilution, carbide morphology, defects, thermal transport, or strain tolerance. Other marine and naval studies document metallurgical degradation, thermal-spray protection, residual fuel corrosion, and limitations of historical valve-alloy selection [6,7,8,9] (Tier A/B).

Modern failure analyses reinforce this coupled interpretation. Heavy-duty diesel exhaust valves have failed through hot corrosion, corrosion fatigue, microstructural instability, thermo-mechanical stress concentration, and progressive loss of hot strength [10,11,12,13,14,15] (Tier B). The final fracture surface often records only the last stage of a longer degradation sequence. Laboratory studies, by contrast, often isolate one variable using flat coupons or automotive-scale components. These tests are valuable for identifying mechanisms, but their wear rates and lives cannot be transferred directly to medium-speed marine valves. Such transfer requires matched contact geometry, thermal gradients, impact dynamics, deposit chemistry, and cooling conditions.

Existing reviews cover high-temperature diesel materials, corrosion-resistant hardfacings, and high-temperature wear of laser-clad Ni-based coatings [16,17,18]. What remains missing is a valve-specific decision structure linking service boundaries and sealing heat rejection to the substrate, working layer, process defects, inspection evidence, and repair history. This review addresses that need through condition-qualified comparisons, an explicit evidence register, and a worked qualification matrix. Coupled degradation is not presented as a new discovery; recent multiphysics modeling already links oxide evolution, temperature, and wear [19]. Instead, the framework defines which evidence can be transferred, where transfer fails, and what validation is required before material, repair, or life management decisions.

## 2. Review Scope and Evidence Method

This review organizes evidence by transferability and mechanism rather than as a catalog of materials and processes. Quantitative values are retained only with their test basis and are not pooled across non-equivalent endpoints, thermal histories, contact geometries, or environments. Section 3 defines valve-specific service boundaries, and Section 4 examines coupled degradation. Section 5 and Section 6 assess bulk materials, structural design, surface engineering, and remanufacturing. Section 7 evaluates testing, monitoring, and prognosis. Section 8 and Section 9 translate the evidence into a qualification framework and prioritized research agenda. This structure separates evidence synthesis from service life claims and makes transfer boundaries explicit.

This is a reference-seeded structured narrative review, not a systematic review or meta-analysis. The original manuscript contained 117 references but no recoverable database export or screening log. Historical identification and exclusion counts therefore could not be reconstructed. On 12 September 2026, we conducted a bounded update through the Crossref Works API. We searched articles published from 1 January 2024 to 12 September 2026, sorted results by relevance, and retained the first 20 records per query. The seven queries were “marine diesel exhaust valve failure”; “marine fuel methanol ammonia engine corrosion deposits”; “exhaust valve alloy oxidation hot corrosion”; “Stellite laser cladding thermal fatigue valve”; “thermal spray coating hot corrosion valve”; “valve remanufacturing additive manufacturing repair”; and “marine engine valve leakage condition monitoring digital twin”. Publisher pages and accessible full texts supported targeted verification. These checks were not independent exhaustive database searches.

**Figure 1:**
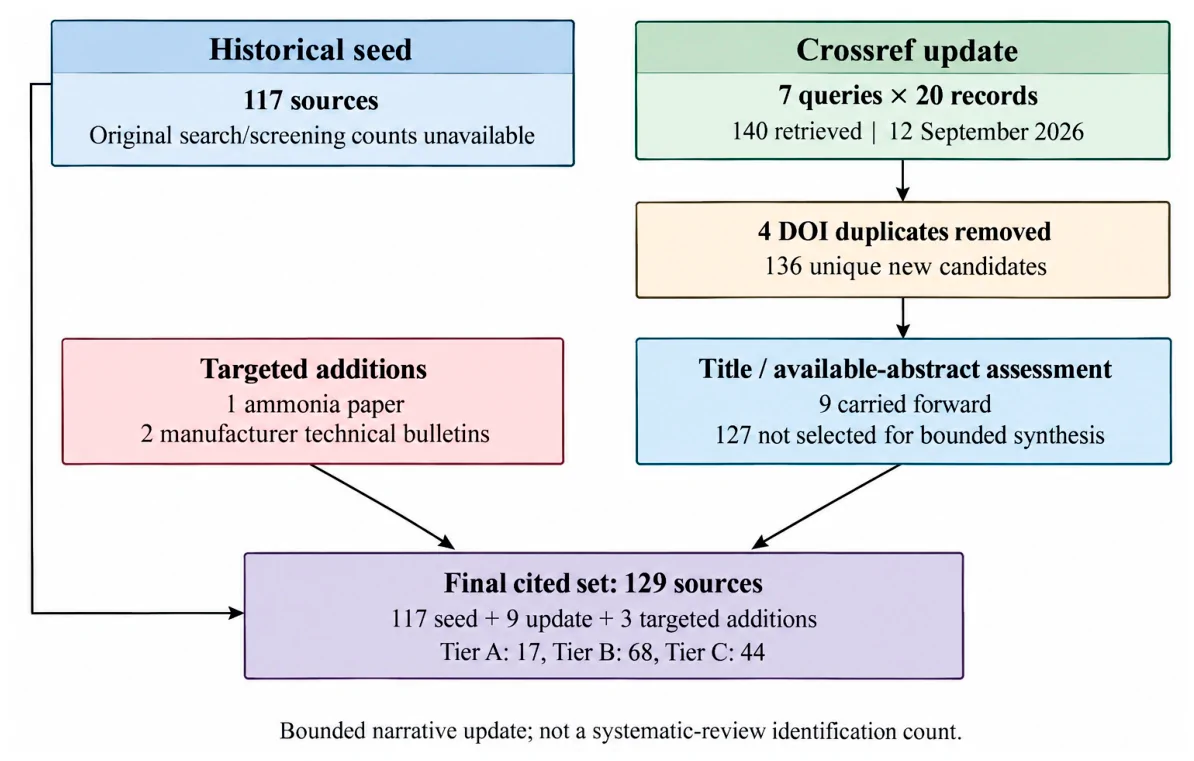
Literature search and study selection flow for the bounded September 2026 update, from the 117-reference seed set to the final set of 129 sources. Arrows show flow; colors distinguish source sets and screening stages.

The update retrieved 140 records. DOI deduplication removed four records already present in the seed set or another query, leaving 136 candidates. Screening of titles and available abstracts retained nine sources relevant to the defined mechanism, fuel transition, process, or monitoring questions. The remaining 127 records were not carried forward. Targeted publisher searches added one Nimonic 80A–ammonia study, and two manufacturer bulletins supplied missing thermal and stress rupture data. The final set comprised 129 cited sources: 117 seed references and 12 additions. Eligibility required a valve-relevant mechanism, material, process, diagnostic method, or explicit transfer boundary. We excluded unrelated equipment, non-metal repair, and coatings without a useful comparison. Selection was qualitative and not independently duplicated. We did not pool effect sizes or assign a formal risk-of-bias score. The search cap, relevance ranking, incomplete full-text access, and retained seed set preclude claims of comprehensive coverage. Figure 1 reports only the update. Table 1 defines the evidence tiers used in the narrative synthesis. Supplementary Tables S1–S3 record retrieved records, screening decisions, evidence tiers, and quantitative extraction notes.

Figure 1 provides the auditable flow of the literature-search update. The 117 seed references are shown separately because their earlier screening history is unavailable.

## 3. Valve Architecture and Service Boundary Conditions

A medium-speed exhaust valve comprises coupled functional zones under nonuniform service conditions. Material or repair comparisons must specify thermal gradients, contact state, impact frequency, microslip, gas and deposit chemistry, and heat rejection paths. These quantities differ across the combustion face, seat band, head–neck transition, stem–guide pair, and tip. Peak head temperature or engine speed alone cannot represent these local demands. The following subsections define the boundary conditions used in later mechanism and material comparisons.

### 3.1. Functional Zones and Heat Rejection Paths

The valve should be divided into functional zones rather than treated as a uniform component. The combustion face absorbs unsteady gas heat flux and pressure. The fillet and neck convert head bending into multiaxial stress and frequently contain the steepest section changes. The seating face provides the gas seal and a major intermittent heat path to the cooled seat. The stem carries axial load, guides motion, transfers heat to the guide, and experiences mixed lubrication or boundary friction. The tip and keeper grooves concentrate contact and assembly stresses. Each zone therefore requires a different balance of strength, toughness, thermal conductivity, oxidation resistance, hardenability, machinability, and dimensional stability.

**Figure 2:**
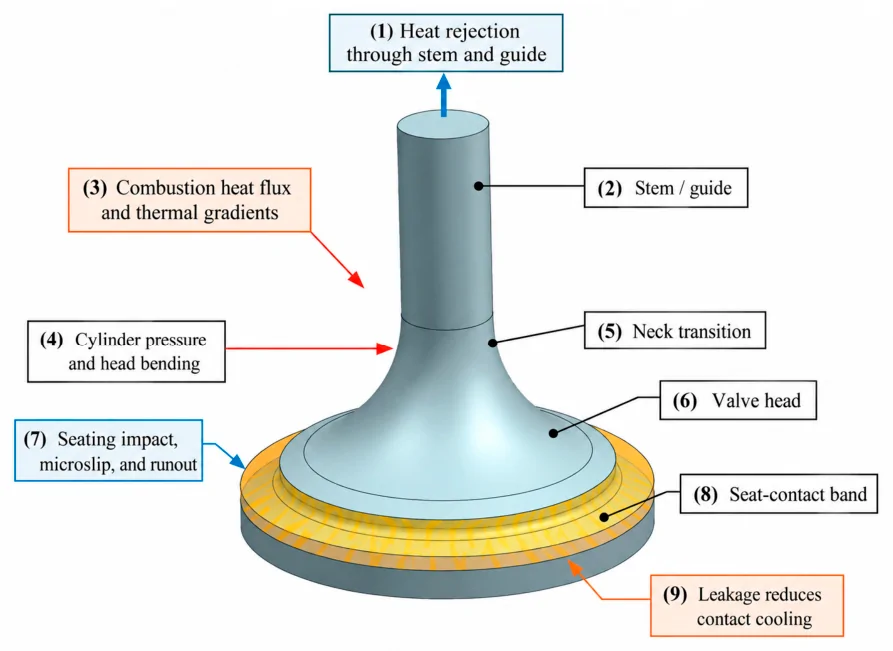
Functional zones, coupled service loads, and principal heat rejection paths of a medium-speed diesel exhaust valve.

Figure 2 maps these functional zones to the thermal, mechanical, tribological, and chemical loads that must be considered together.

Heat leaves the valve through the seated contact and through the stem/guide, while hot gas supplies the spatially and temporally varying flux. Transient heat transfer and contact studies show that gas-side coefficients, seat conductance, contact duration, geometry, and local pressure must be specified [20,21,22,23,24] (Tier B). A wider nominal contact band can increase the conductive area, but pressure-dependent conductance, deposits, runout, and incomplete circumferential contact can change that trend. Table 2 distinguishes the model predictions and indirect thermometry used for the temperature examples in this review.

**Figure 3:**
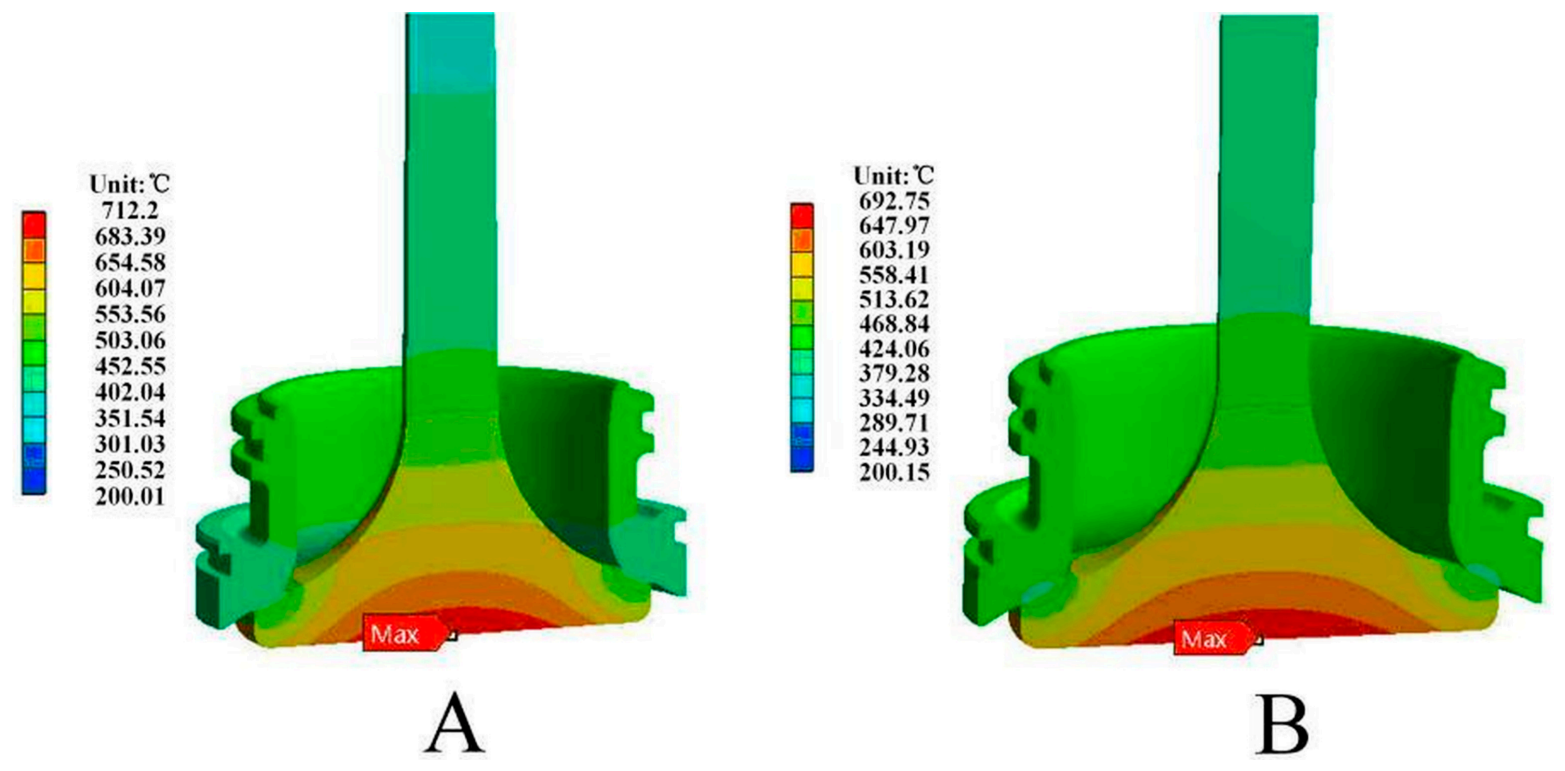
Predicted temperature fields and peak temperature difference between alternative marine exhaust valve designs [2]. (**A**) With welded facing; (**B**) without welded facing. Source: [2], CC BY 3.0.

The model comparison reproduced in Figure 3 illustrates how an apparently local change at the valve–seat interface can alter the full-head temperature field.

A facing can improve wear or corrosion resistance while increasing thermal resistance. Its net effect depends on which surface is coated, the local heat flow direction, contact quality, and substrate conduction. The 19 °C difference in Figure 3 cannot be attributed to thickness alone because material and structural choices also changed. Section 6.4 therefore separates a controlled analytical thickness/conductivity sensitivity from the source-specific finite-element results.

### 3.2. Mechanical Loading, Seating Impact, and Microslip

Cylinder pressure bends the valve head while the spring, actuator, guide, and seat constrain its motion. During closing, valve mass and velocity generate an impact impulse whose distribution depends on face angle, seat width, eccentricity, local stiffness, surface waviness, and valve rotation. The contact is not purely normal. Elastic head deflection under cylinder pressure produces radial relative motion along the conical sealing interface, and runout produces a circumferentially nonuniform load. Even micrometer-scale sliding becomes consequential at hundreds of millions of cycles.

**Figure 4:**
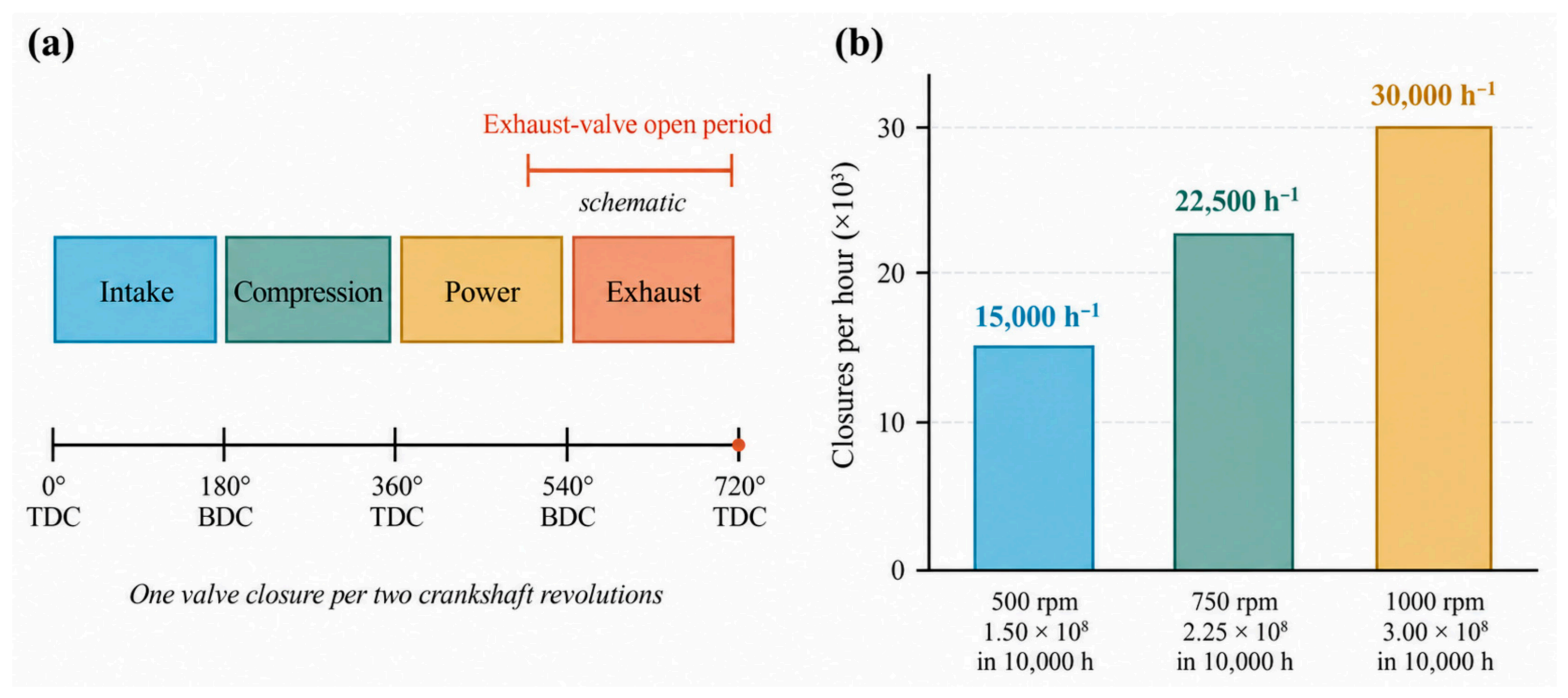
Four-stroke exhaust valve cycle and seating exposure: (**a**) events over 720° crank angle; (**b**) closures per hour and cumulative closures over 10,000 h at representative medium-speed operating speeds.

Figure 4 converts the four-stroke event sequence into seating exposure at representative medium-speed operating speeds; the calculation emphasizes why extremely small per-event damage increments become important in long service.

Diesel and heavy-duty valve studies identify adhesive transfer, plowing, abrasion by detached oxides, oxidative wear, plastic flow, compaction, and surface-fatigue spalling as concurrent contributors to recession [26,27,28,29]. Direct measurements and simulations quantify sealing-interface sliding, while tribofilm studies show that oil- and ash-derived residues can temporarily separate metal surfaces [30,31]. Consequently, a static Hertzian calculation is necessary but insufficient: the relevant state includes impact energy, cyclic tangential displacement, temperature-dependent material response, oxide or tribofilm condition, and evolving geometry (Tier B).

Wear response is often non-monotonic with temperature. Iron–nickel and nickel-base valve alloys can form mixed oxide layers that lower adhesion under some conditions, yet impact or thermal cycling can fragment those layers into abrasive third bodies [32]. Recent impact-wear studies emphasize the roles of contact load, face angle, material pairing, and surface processing [33,34,35]. Recession models and laboratory impact–sliding tests are most useful when calibrated against measured contact widths and failure morphologies rather than a single mass-loss value [36,37,38,39] (Tier B).

### 3.3. Chemical Environment, Deposits, and Fuel Dependence

Oxidation, sulfidation, vanadate fluxing, and erosion–corrosion should be distinguished. Oxidation forms an oxide scale; a coherent scale can protect the alloy. Sulfur ingress can instead form internal sulfides and locally consume chromium. Vanadium- and sodium-bearing deposits can form liquid salts that dissolve or disrupt protective oxides; their activity depends on salt composition, oxygen/sulfur potentials, and temperature. Particle impact, sliding, or leakage flow can then remove the weakened scale and expose fresh metal. These pathways can coexist, but neither a universal melting temperature nor a corrosion rate follows from the abbreviation V–Na–S [8,9,40,41] (Tier B, valve/environment analogs). Table 3 separates fuel-derived expectations from measured exposure, Table 4 links valve zones to dominant loads and diagnostic evidence, and Table 5 retains the conditions behind quantitative examples.

**Figure 5:**
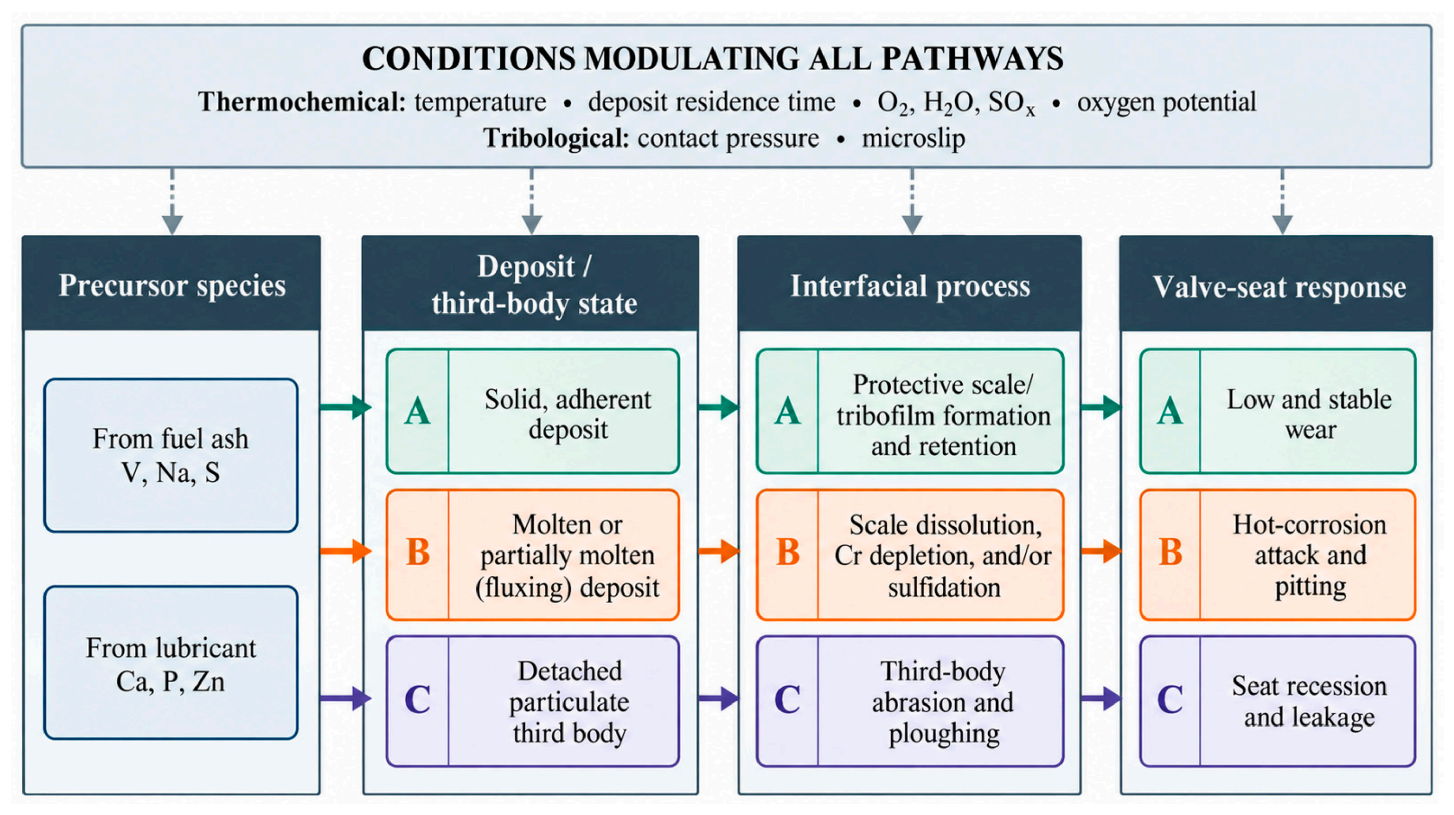
Conditional pathways linking fuel- and lubricant-derived species to deposits, scale or tribofilm evolution, and valve seat damage. Solid arrows show pathways A–C; dashed arrows show modulation; colors identify the three pathways.

Figure 5 summarizes the conditional pathways by which the same fuel-, lubricant-, and gas-derived species may either support a protective film or accelerate corrosion and third-body wear.

ISO 8217:2024 and IMO sulfur requirements provide fuel specifications and regulatory context, not a prediction of deposit phase or valve life [43,44] (Tier C). Residual fuel, distillate, LNG, methanol, and ammonia should therefore be compared using actual fuel/pilot-fuel composition, lubricant carry-over, contaminants, and near-wall conditions. A high-speed marine methanol–diesel CFD study, for example, addresses combustion and emissions under changing methanol energy substitution and timing; it does not measure medium-speed valve corrosion [48] (Tier A, different speed class and endpoint). Ammonia evidence must likewise distinguish hot Ni80A nitridation from lower-temperature aqueous or condensate attack on cast-iron liners [49,50] (Tier C).

## 4. Coupled Degradation and Failure Mechanisms

The dominant degradation modes are strongly coupled in service. Thermal gradients and mechanical restraint create cyclic strain; oxidation and deposits alter the surface chemistry and roughness; repeated impact and microslip change the sealing geometry; and loss of sealing can modify heat flow and local gas exposure. For this reason, the mechanisms below are separated for analysis but interpreted as interacting pathways rather than independent failure categories. The central distinction is between directly observed damage signatures and the hypothesized feedback that connects them over time.

### 4.1. Thermo-Mechanical Fatigue and Thermal Shock

Thermo-mechanical fatigue (TMF) arises because the head surface heats rapidly while the cooler interior, seat, stem, and cylinder head restrain expansion. The stress reverses or relaxes as the load changes and the valve opens, closes, and cools. Local tensile strain is intensified at the seat edge, neck fillet, hardfacing termination, weld interface, machining marks, and pits. Start–stop cycles and rapid load changes can be more damaging per cycle than steady operation, while high-cycle seating loads propagate small environmentally assisted cracks between major thermal transients. This interpretation is an authors’ synthesis and not an independently validated service life rule.

A first screening relation is Δ*ε_th_* ≈ *α*Δ*T*. For α = 15 × 10^−6^ K^−1^ and ΔT = 300 K, the free thermal-strain range is 0.0045 (0.45%). A biaxially restrained elastic estimate, $\Delta \sigma_{\mathrm{el}}\approx \frac{\mathrm{\chi E\alpha}\Delta T}{1-\nu }$, gives 964 MPa for E = 150 GPa, ν = 0.30, and full restraint χ = 1. The corresponding amplitude is 482 MPa. For χ = 0.25, the estimated range and amplitude are 241 and 121 MPa, respectively. These values are illustrative, not measured valve stresses. Yielding, creep relaxation, nonuniform temperature, and phase lag may invalidate the elastic estimate.

The strain–life relation $\varepsilon_{a}=\left(\frac{\sigma_{f}^{\prime }}{E}\right){\left(2N_{f}\right)}^{b}+\varepsilon_{f\left(2N_{f}\right)}^{\prime c}$ defines *ε_a_* as strain amplitude and *N_f_* as cycles to crack initiation. The coefficients require measurements for the specific alloy, temperature, phase relation, and environment; none are fitted here. The dwell term $D_{c}=\sum \left(\frac{t_{\mathrm{dwell}}}{t_{r\left(T,\sigma \right)}}\right)$ distinguishes time-dependent creep, where *t_r_* is rupture time at the relevant temperature and stress. Combining *D_c_* with fatigue damage remains a screening approximation until interaction and oxidation effects are calibrated. Manufacturer data for directly aged Inconel 751 report 100 h rupture stresses of 476 MPa at 649 °C and 193 MPa at 816 °C [52] (Tier C). These values are neither fatigue stresses nor marine valve allowables.

The available 42Cr9Si2 data illustrate the danger of substituting isothermal fatigue for TMF: rotating-bending strength at 10^7^ cycles falls from approximately 569 MPa at 400 °C to 300 MPa at 650 °C [53] (Tier B). The test does not reproduce start–stop phase lag or high-temperature dwell. Component testing should therefore record heating/cooling rates, gradient, hold time, mechanical constraint, and the crack initiation criterion as well as cycle count.

**Figure 6:**
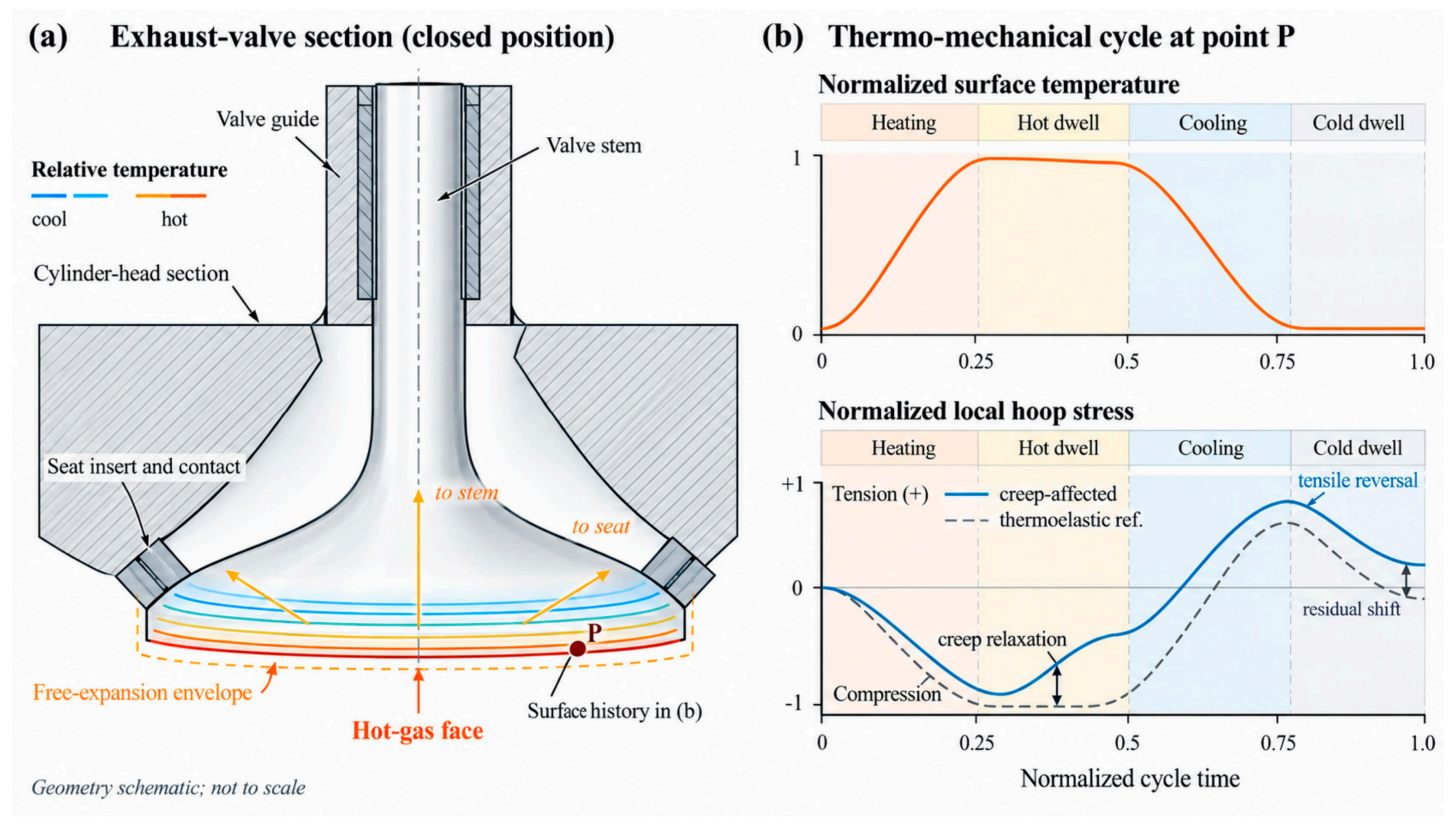
Schematic thermo-mechanical fatigue cycle of an exhaust valve head: (**a**) transient thermal gradient and constrained expansion; (**b**) qualitative surface-strain evolution during heating and cooling.

The qualitative cycle in Figure 6 distinguishes the through-head thermal gradient from the constrained surface-strain reversal that drives TMF crack initiation.

### 4.2. Oxidation, Hot Corrosion, and Microstructural Degradation

Cr-containing Fe- and Ni-base valve alloys rely on adherent Cr_2_O_3_, spinel, and, where chemistry permits, Al-rich scales. Their benefit depends on scale continuity, growth stress, adhesion, and the supply of Cr or Al from the substrate. Cyclic oxidation studies on valve-related alloys show that scale spallation and renewed oxidation can consume the protective reservoir and roughen the contact surface [45,54]. High-temperature tribology further shows that an oxide can be either protective or abrasive depending on its cohesion and retention [55] (Tier B/C).

In V–Na–S environments, molten deposits can flux the scale and create an erosion–corrosion surface whose pits become fatigue initiators [56]. Laser-welded and thermally sprayed systems have therefore been evaluated specifically for large-diesel or simulated hot-corrosion exposure [57,58]. Ceramic or metallic barrier concepts can slow deposit access, but open porosity, segmentation cracks, or edge defects may become short paths for molten salts; conventional thermal barrier systems can also accumulate harmful vanadium products [59,60]. Classical work on valve burning likewise links scale failure and localized chemistry to rapid material loss [61,62] (Tier B/C).

The substrate evolves at the same time. Cr–Mn–N austenitic valve steels can undergo carbide precipitation, cellular decomposition, grain boundary chromium depletion, and loss of toughness. Ni-base alloys can overage, coarsen strengthening precipitates, and develop grain boundary damage. Surface hardness may decrease because of tempering, carbide redistribution, oxidation-assisted depletion, or dilution. Thus, hardness mapping should be interpreted with microstructure and chemistry: a hard but continuous brittle carbide network can lower thermal fatigue resistance, whereas moderate retained hardness in a tough, well-bonded layer may give longer service. This interpretation is an authors’ synthesis and not an independently validated service life rule.

### 4.3. Impact–Sliding Wear, Recession, and Sealing Loss

Valve seat recession is a geometric state variable produced by several interacting mechanisms rather than a single wear mode. Impact can compact asperities and drive radial plastic flow; microslip promotes adhesion and plowing; detached oxides or deposits act as third-body abrasives; cyclic oxidation repeatedly creates and removes material; and subsurface cyclic stress can initiate delamination. Classic impact studies, finite-element simulations, hollow-head valve tests, and oxidized-alloy comparisons therefore indicate that contact angle, mass, velocity, stiffness, hot hardness, oxide stability, and material pairing must be evaluated jointly [25,63,64,65] (Tier B). Because recession changes contact width, local pressure, and the heat rejection path, it can also modify the conditions that govern its subsequent evolution.

Fretting predictions and recent valve cone plasma treatments provide useful mechanism-scale data but remain Tier B/C until validated on the target engine [66,67]. Likewise, high-temperature NCF3015 tribology and Stellite 12 impact tests support interpretation of alloy response and oxide film stability rather than a universal service interval [68,69]. Field-derived tribofilms differ from laboratory films because they integrate real lubricant, fuel, load, temperature, and replenishment histories [42]. Accordingly, a useful recession model should evolve contact width, pressure, microslip, temperature, film/deposit state, and leakage together. Experimental studies should report the circumferential wear profile and contact band evolution in addition to mass or volume loss, because identical bulk loss can correspond to very different sealing consequences [24,36,37,66].

### 4.4. Leakage, Guttering, and Fracture Cascade

Loss of gas tightness can couple thermal and mechanical damage, but the full chronology is not directly demonstrated by a single longitudinal dataset. Marine recovered-valve examinations establish coexisting face loss, fatigue propagation, and terminal fracture [3,4] (Tier A, post-mortem). Experiments with imposed marine valve leakage establish changes in combustion and exhaust response [70] (Tier A, engine test). The inference that a growing leak continuously raises a particular local metal temperature and recession rate requires time-resolved contact, heat flux, and material loss measurements that these studies do not provide. Table 6 distinguishes observed links from the proposed feedback closure.

**Figure 7:**
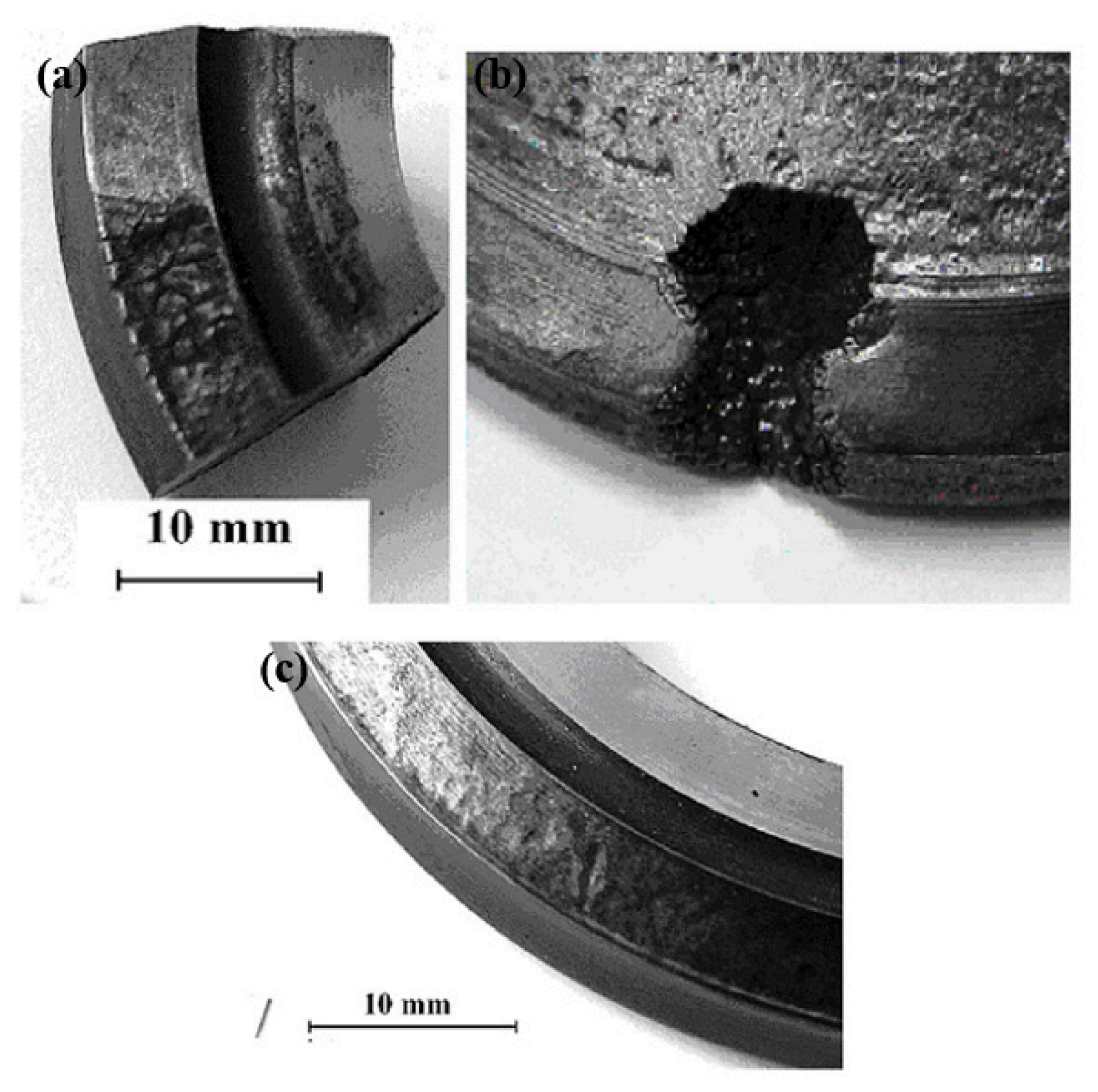
Representative marine exhaust valve face damage: (**a**) corrosion-related pavement morphology; (**b**) severe facing and substrate loss associated with leakage and hot-gas attack; (**c**) repairable face wear [4]. Source: [4], CC BY 4.0.

The field morphologies in Figure 7 illustrate distinct damage states, including limited wear and severe loss of facing and substrate. They are not successive observations of the same valve.

**Figure 8:**
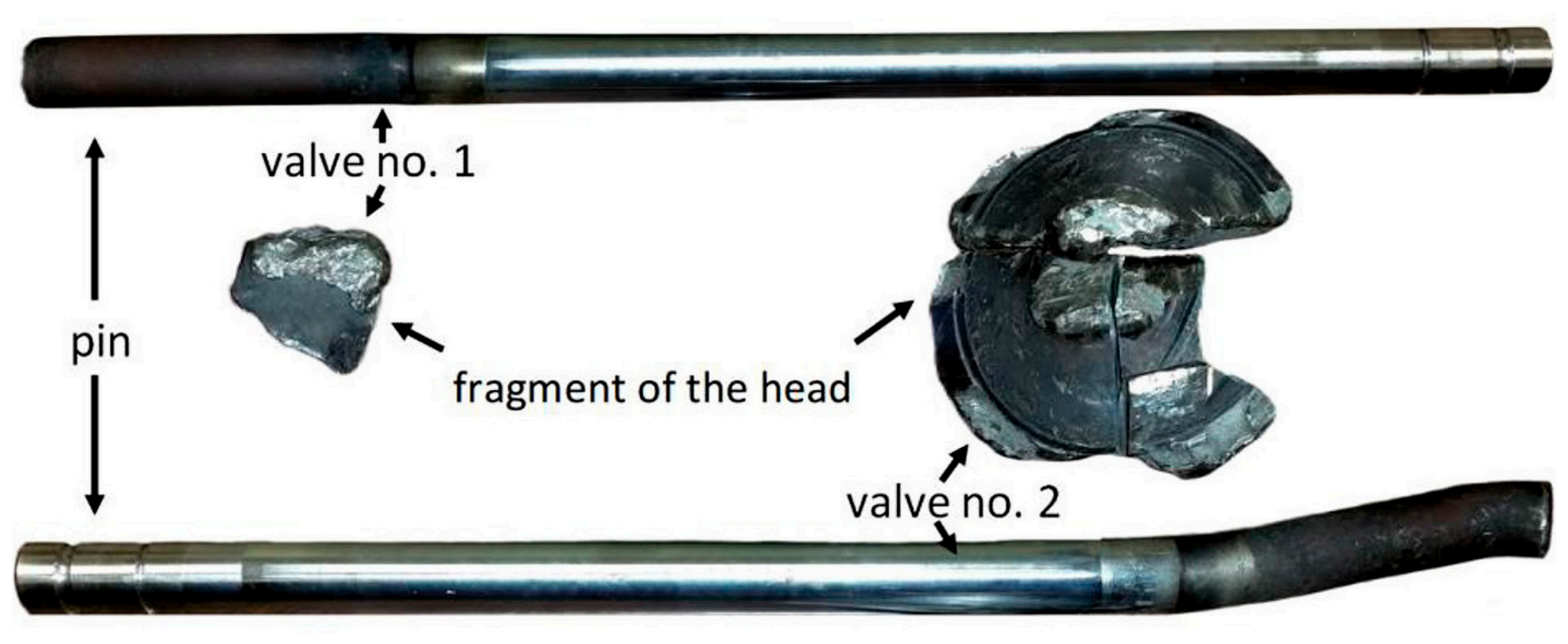
Recovered exhaust valve fragments from a Stork–Wärtsilä 6SW280 engine, showing stem separation, head fragmentation, and severe head deformation [3]. Source: [3], CC BY 4.0.

Recovered components from the 6SW280 casualty are shown in Figure 8; they demonstrate how a primary head separation can create severe, morphologically different secondary damage.

**Figure 9:**
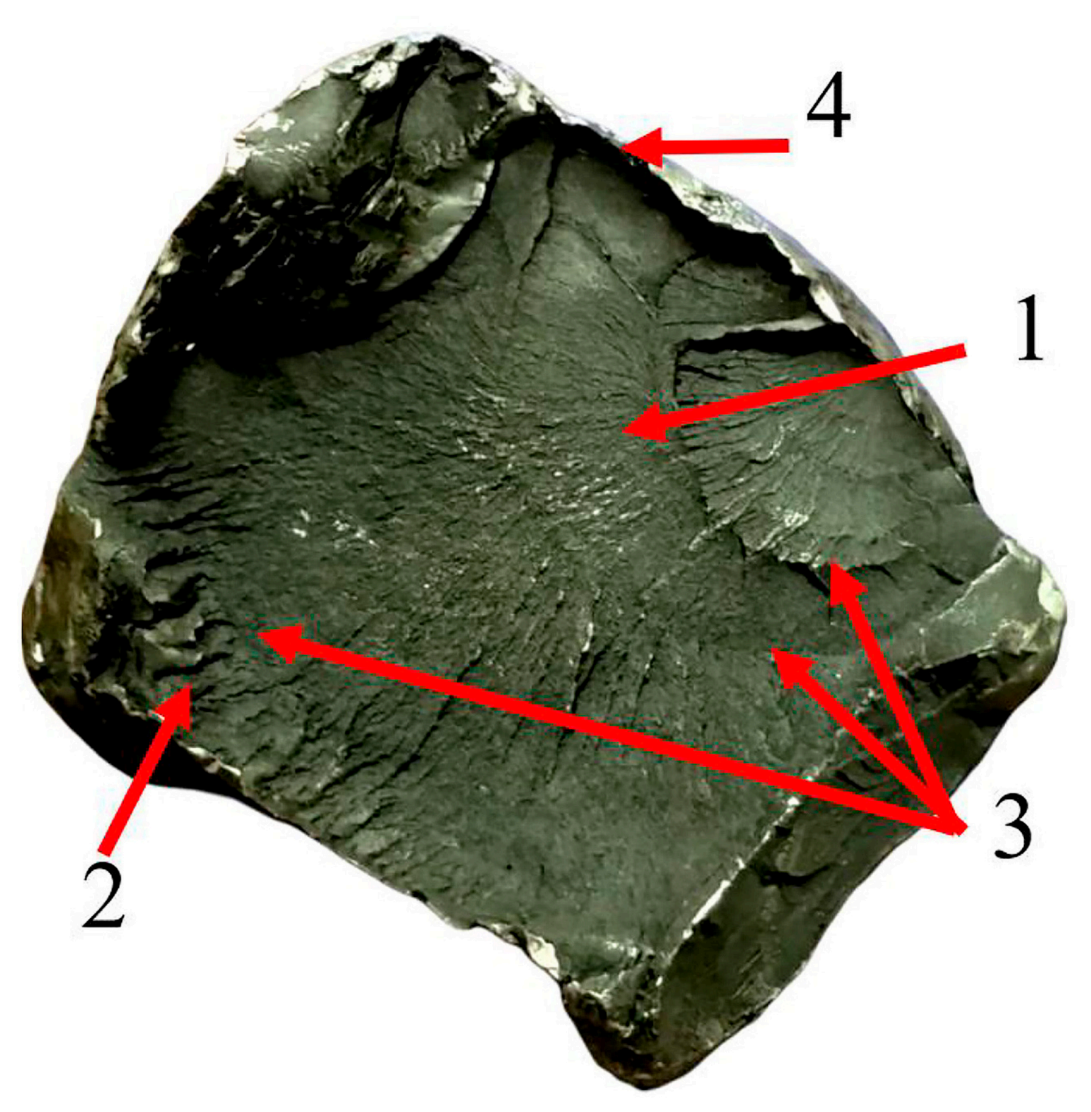
Fracture surface of the first failed exhaust valve head from a Stork–Wärtsilä 6SW280 engine: (1) fatigue propagation region; (2) final fracture; (3) crack-front markings; (4) peripheral plastic deformation [3]. Source: [3], CC BY 4.0.

Figure 9 isolates the fracture surface of the first failed head and separates its progressive fatigue region from the terminal overload and peripheral deformation.

The fracture photograph captures why the first failed component must be distinguished from secondary damage. Crack-front markings indicate progressive fatigue, while the rougher final region and peripheral deformation record the terminal event. After fragment release, impacts can create entirely different fracture morphologies on the paired valve and downstream components. Failure reports should preserve both the spatial evidence and the engine event log before cleaning or sectioning; otherwise, the initiating mechanism may be confused with post-release collision damage. This interpretation is an authors’ synthesis and not an independently validated service life rule.

**Figure 10:**
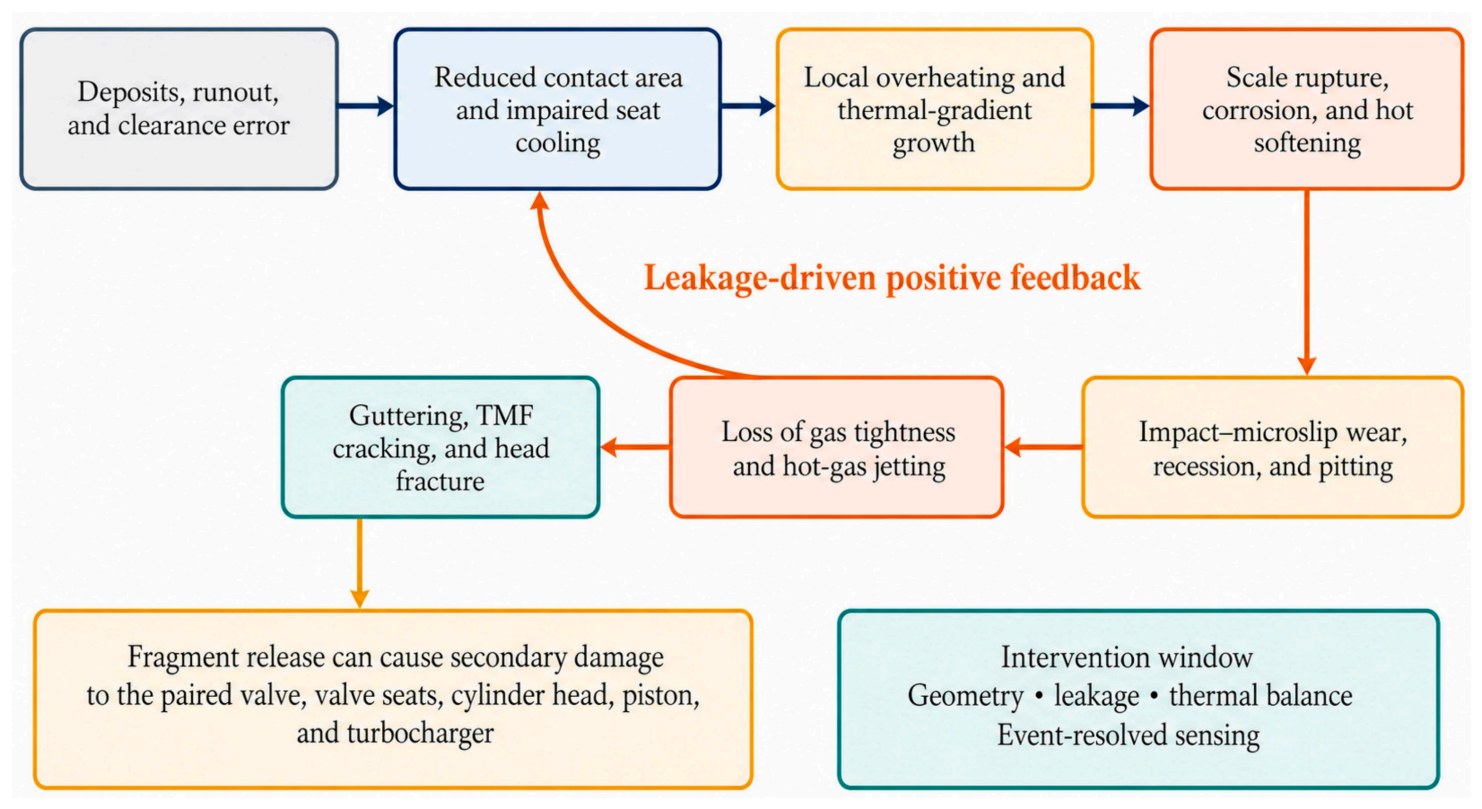
Proposed leakage–damage feedback pathway for marine exhaust valves, synthesized from the available evidence. Arrows show direction; orange marks feedback; colors identify precursor and intervention states.

Figure 10 assembles a hypothesized feedback pathway consistent with the separately observed damage signatures. The diagram does not establish a universal order of events; corrosion or fatigue can precede leakage, and fracture can occur without a documented guttering stage.

Within the proposed coupling, the nominal contact area is *A_c_* = 2*πrbφ*, where r is mean seat radius, b is contact width, and φ is the intact circumferential fraction. The cycle-averaged seat conductance is $G_{s}=\frac{f_{cA_{c}}}{\frac{1}{h_{c}}+\frac{t_{c}}{k_{c}}}$. Here, *f_c_* is the closed fraction, *h_c_* is contact conductance per unit area, and $\frac{t_{c}}{k_{c}}$ is facing resistance. A lumped steady balance gives $T_{w}=\frac{Q_{g}+Q_{\mathrm{jet}}+G_{sT_{s}}+G_{oT_{o}}}{G_{s}+G_{o}}$. The terms represent gas heat input *Q_g_*, leakage heat input *Q_jet_*, seat temperature *T_s_*, and conductance *G_o_* to temperature *T_o_*. Holding other terms fixed isolates sensitivity but does not reproduce the three-dimensional valve temperature field. Narrowing b also changes contact pressure and possibly *h_c_*, so inverse-width scaling remains conditional.

For a small leakage slot, *A_l_* ≈ *L_lg_*, where *L_l_* is open-arc length and g is gap height. Ideal-gas flow becomes choked when $\frac{p_{b}}{p_{0}}\leq {\left[\frac{2}{\gamma +1}\right]}^{\frac{\gamma }{\gamma -1}}$. The corresponding mass flow is

$$\dot{m}=C_{dA_{lp_{0\sqrt{\frac{\gamma }{RT_{0}}{\left[\frac{2}{\gamma +1}\right]}^{\frac{\gamma +1}{2\left(\gamma -1\right)}}}}}}.$$

Here, *p*_0_ and *T*_0_ are upstream stagnation conditions; *p_b_* is downstream pressure; R is the specific gas constant; γ is the heat-capacity ratio; and *C_d_* is the discharge coefficient. For *p*_0_ = 2.0 MPa, *p_b_* = 0.20 MPa, *T*_0_ = 1000 K, γ = 1.33, R = 287 J kg^−1^ K^−1^, *C_d_* = 0.70, *L_l_* = 10 mm, and g = 10 µm, the slot area is 0.10 mm^2^. The ideal mass flow and throat velocity are approximately 0.176 g s^−1^ and 572 m s^−1^, respectively. Doubling the gap doubles ideal mass flow at fixed upstream conditions, but not sonic velocity. Real cyclic leakage requires time-dependent pressure and calibrated compressible-flow discharge.

Leakage heating may be represented as $Q_{\mathrm{jet}}=\eta \dot{m}c_{p\left(T_{0}-T_{w}\right)}$, where η is the unknown local heat deposition fraction. A schematic recession relation is $\frac{\mathrm{dr}}{\mathrm{dN}}=\frac{\left[\frac{K_{wF_{\mathrm{ns}}}}{H\left(T\right)}+V_{\mathrm{impact}}+V_{\mathrm{chem}}\right]}{A_{w}}$. Here, N is seating-cycle count, *F_n_* is normal force, s is microslip per cycle, H is hot hardness, *K_w_* is a calibrated sliding coefficient, and *A_w_* is worn area. *V_impact_* and *V_chem_* are impact and chemical loss volumes per cycle. Time-based corrosion must be converted using the actual exposure per cycle. These equations connect contact, leakage, temperature, and loss, but no guttering coefficient is inferred from unrelated coupon mass loss. The illustrative velocity and temperature drop are therefore not converted into recession rate or life (authors’ proposal informed by Tier B models [24,36,37,66]).

## 5. Bulk Materials and Structural Design

Bulk material selection defines the temperature-dependent strength, fatigue, creep, oxidation, and manufacturing envelope within which the valve must operate, but the grade name alone does not determine durability. Heat treatment, forging history, microstructural stability, section architecture, and the location of dissimilar joints can be as important as nominal composition. The comparison in this section therefore emphasizes property retention and structural placement under service-relevant temperatures rather than room-temperature strength or alloy cost in isolation.

### 5.1. Martensitic and Austenitic Valve Steels

Martensitic Cr–Si valve steels combine strength and processability, but their useful temperature range must be judged from the actual heat treatment and loading. For quenched-and-tempered 42Cr9Si2, reported ultimate/yield strengths decrease from 1096 ± 16/901 ± 19 MPa at 25 °C to 512 ± 12/454 ± 15 MPa at 650 °C; elastic modulus decreases from 222 ± 18 to 137 ± 9 GPa [53] (Tier B, valve-steel tests). These measured trends support temperature-specific qualification; they do not establish a common allowable head temperature for every martensitic grade.

Cr–Mn–N austenitic steels such as 21-4N and 23-8N are widely used for exhaust valve heads because N solid-solution strengthening, stable austenite, hot strength, oxidation resistance, and weldability provide a useful cost–performance balance. Material surveys, fatigue tests, and ultrasonic surface rolling studies quantify their composition and the scope for surface-induced fatigue improvement [71,72,73]. Their long-term weakness is not simply bulk softening: cellular M_23_C_6_ precipitation, grain boundary coarsening, Cr depletion, and environmental cracking can reduce ductility and scale renewal. Heat treatment and prior service history must therefore accompany the grade designation (Tier B).

**Figure 11:**
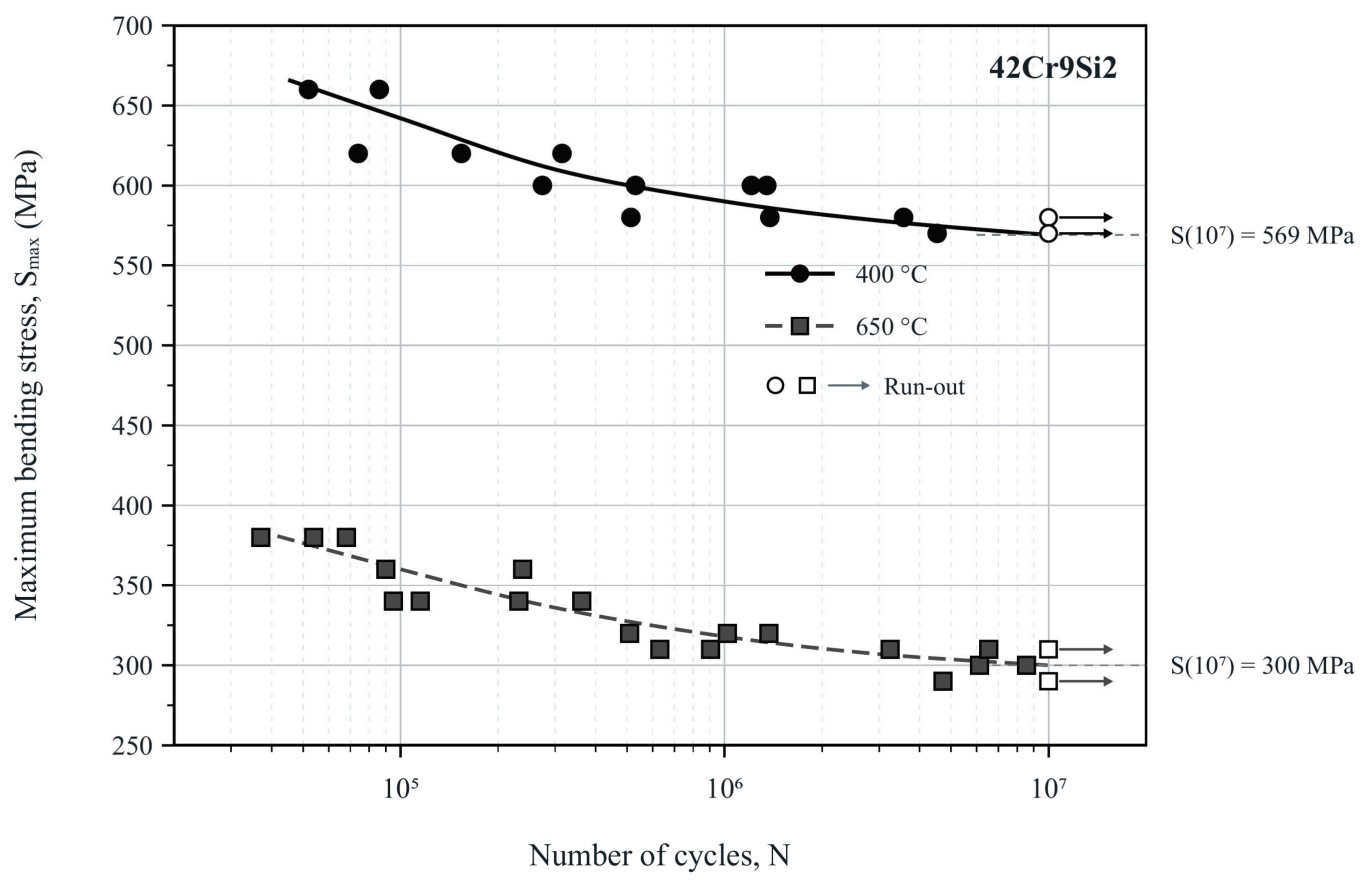
S–N curves of 42Cr9Si2 valve steel at 400 and 650 °C; the reported 10^7^-cycle strength decreases from approximately 569 to 300 MPa [53]. Adapted from [53], CC BY 4.0.

The temperature sensitivity of a martensitic valve steel is quantified by the S–N data in Figure 11: the reported 10^7^-cycle strength of 42Cr9Si2 is substantially lower at 650 °C than at 400 °C.

### 5.2. Fe–Ni and Ni-Base Alloys

Fe–Ni alloys such as NCF3015 and precipitation-strengthened Ni-base alloys such as Nimonic 80A and Inconel 751 provide different combinations of hot strength, oxidation resistance, thermal response, manufacturability, and cost. Valve material wear and forging studies support these distinctions [32,68,74] (Tier B), while manufacturer data provide additional condition-specific thermal and stress rupture values [52,75] (Tier C, Table 7). A higher Ni content or higher hot strength should therefore not be treated as an automatic ranking criterion: thermal expansion, conductivity, creep relaxation, forgeability, weld/repair compatibility, and environmental response all affect the local stress–temperature state. Nimonic 80A, for example, can develop internal sulfides in simulated residual fuel deposits and nitridation in an ammonia-rich atmosphere [8,49]. Alloy substitution consequently requires a matched exposure and processing route rather than a blanket claim of superior corrosion resistance.

Valve-specific process studies illustrate these constraints. Closed-die forging of Nimonic 80A requires control of dynamic recrystallization and microstructural uniformity [76,77]. NCF3015 forgings can develop intergranular cracking and intermediate-temperature embrittlement if carbide precipitation, cooling, and deformation are poorly controlled [74]. Gas-engine analogs show that even Inconel-class valves fail prematurely when seating, guide condition, clearance, or deposits impose the wrong thermal boundary [46]. Material selection defines damage tolerance; seating integrity and heat transfer architecture define applied demand (Tier B).

### 5.3. Bimetallic, Compound, and Hollow Sodium-Cooled Architectures

Bimetallic valves place a high-temperature alloy in the head and a less expensive, machinable steel in the stem. Inertia or friction welding provides production efficiency, but the thermo-mechanically affected zone contains severe deformation, refined or transformed microstructures, hardness gradients, and residual stress; carbon and alloy-element redistribution can add brittle phases [53,78]. The weld should be kept away from the maximum bending and thermal gradient region, followed by verified post-weld heat treatment and full-circumference nondestructive examination (Tier B).

Compound heads can place a Nimonic-class working layer on a lower-cost substrate [79] (Tier B). Sodium cooling changes the heat transfer architecture, but two results must be separated. Lai et al. compared solid and hollow-stem sodium-filled valves in a gasoline-engine dynamometer test using post-test hardness thermometry, reporting approximately 745 and 590 °C, respectively [25] (Tier B). Their separate hollow-head design showed a 16.1% mass reduction and was evaluated on a component rig. These are different comparisons, and neither directly establishes a marine engine temperature benefit. Friction-weld optimization of 42Cr9Si2 hollow-head valves was investigated subsequently [53]. Cavity integrity, fill ratio, closure welds, and thermal boundaries require validation on the intended engine.

Historical diesel material development confirms that the graded use of expensive alloys is usually more rational than a uniform maximum-grade solution [16,17]. Laser-clad and early compound-valve work similarly established the importance of locating high-performance material only where the demand justifies it [80,81,82] (Tier B/C).

**Figure 12:**
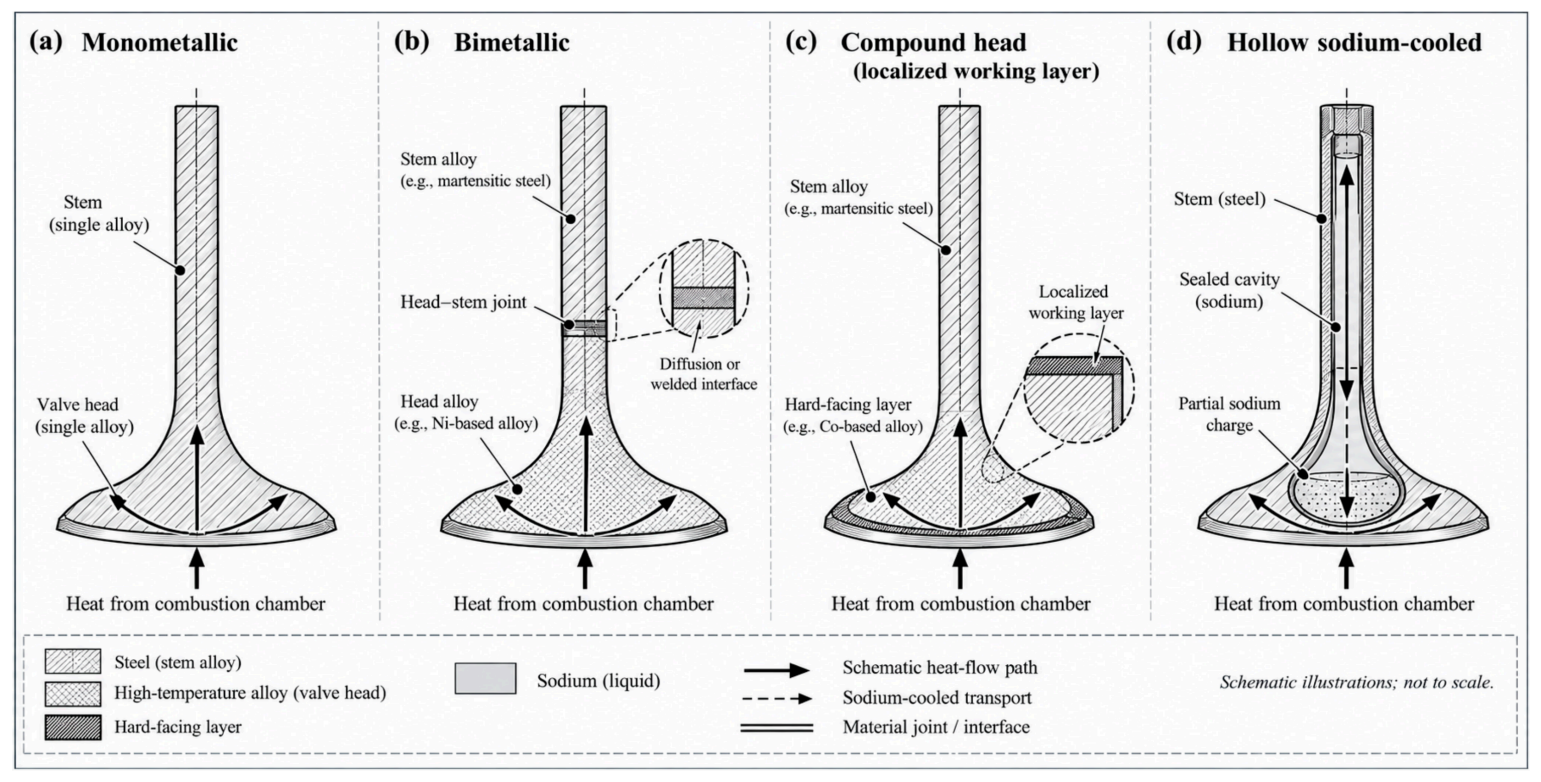
Valve architecture concepts for medium-speed diesel engines: (**a**) monometallic; (**b**) bimetallic with a dissimilar head–stem joint; (**c**) compound head with a localized working layer; (**d**) hollow sodium-cooled design.

The principal architecture options, their critical interfaces, and their intended heat flow paths are compared schematically in Figure 12.

## 6. Surface Engineering and Remanufacturing

Surface engineering changes more than wear resistance. A working layer simultaneously alters hot hardness, oxide/deposit interactions, thermal resistance, residual stress, interface constraint, machinability, and the geometry available for future repair. Remanufacturing adds an additional history dependence because the substrate may already contain thermal exposure, prior heat-affected zones, machining loss, and residual defects. Accordingly, surface-process qualification must evaluate the complete layer–interface–substrate–seat system rather than rank coatings only by nominal composition or hardness.

### 6.1. Wear Resistance and Retention of Sealing Geometry

The seat-contact band needs hot hardness, adhesive-wear resistance, scale stability, toughness, thermal fatigue tolerance, corrosion resistance, machinability, and metallurgical compatibility. These properties conflict. Stellite 6/F uses a comparatively tough Co-rich matrix with Cr-rich carbides and remains a broad-purpose face material. Stellite 12 increases carbide content and high-load wear resistance but reduces strain tolerance and weldability. Stellite 21 or related low-carbon Co alloys favor toughness and anti-galling behavior. NiCrBSi or Colmonoy-type alloys wet and self-flux well and offer corrosion resistance, yet B- and Si-rich hard phases can make the layer cold crack-sensitive. This interpretation is an authors’ synthesis and not an independently validated service life rule.

Hardness is one screening variable, not a sufficient performance ranking. Stellite studies identify carbide continuity, matrix response, oxidation, and dilution as relevant alongside hardness [83,84,85,86] (Tier C). The marine L12 and N facing comparison is particularly informative: post-service near-surface ranges overlap (L12, 473–581 HV5; N, 480–581 HV5), yet the N-facing valve was severely damaged after about 2000 h and L12 showed less damage after about 3000 h [4] (Tier A). These two observations do not establish a controlled life ratio because duty, defects, geometry, and exposure history were not independently matched. They show that comparable measured hardness can accompany markedly different outcomes. Valve-contact laser hardening and recent oxide-addition cladding studies provide further screening examples [87,88] (Tier B/C).

Nitriding and mechanical surface strengthening can be useful where wear depth and subsequent grinding remain within the supported surface zone. Valve-specific nitriding and ultrasonic rolling studies provide relevant evidence [71,89], while textured nitrided coupons are more remote analogs [90] (Tier B/C). In one valve study, nitriding used 580 °C for 35–40 min; an independently comparable case-depth value was not available for this synthesis [25]. A thin case should not be credited with a millimeter-scale repair allowance. Compound-layer integrity, substrate support, and retained dimensions must accompany hardness.

**Figure 13:**
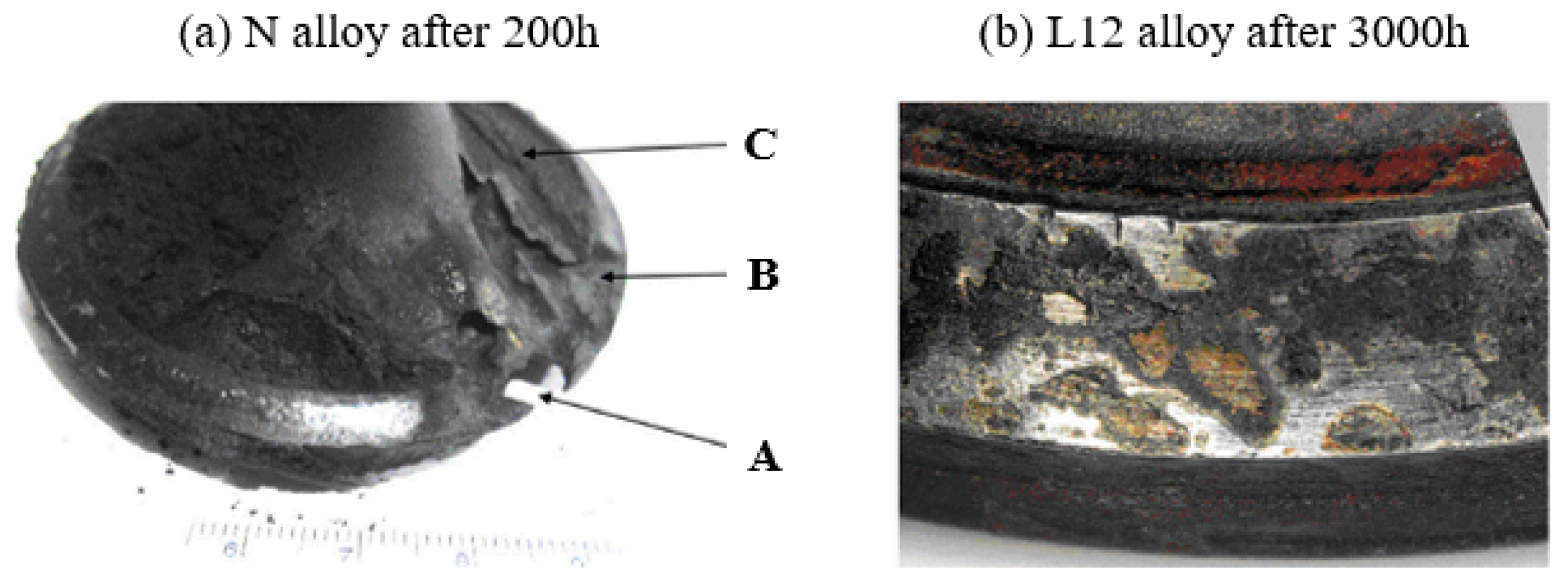
Service degradation of hardfaced valves from a medium-speed marine engine: (**a**) severe loss and fracture of the N-alloy facing after approximately 2000 h; (**b**) less extensive degradation of the L12 facing after approximately 3000 h [4]. In (**a**), A marks the broken section, B lost facing, and C valve-head damage. Source: [4], CC BY 4.0.

The service comparison in Figure 13 reinforces this point: nominally similar cobalt-alloy working layers can develop sharply different damage states because chemistry, solidification structure, defects, dilution, and operating history are not interchangeable.

### 6.2. Oxidation, Hot Corrosion, and Environmental Access

Environmental qualification should first reproduce the relevant salt chemistry, gas atmosphere, temperature window, and deposit residence condition (Table 5). Marine/naval valve-coating work provides application-specific starting points [7] (Tier A), whereas sulfate–vanadate, chloride, or ceramic-barrier coupon studies isolate selected transport and reaction mechanisms [51,58,59,60] (Tier C). Dense metallic coatings can delay chemical ingress, but protection is governed by the connectivity of pores, cracks, unmelted regions, splat boundaries, dilution zones, and exposed edges rather than by nominal composition alone. For HVOF Cr_3_C_2_–NiCr, a recent chloride test reported corrosion-product masses of 3.40 and 10.07 mg cm^−2^ after 100 h at 600 °C for coatings produced from milled and unmilled powder, respectively [51] (Tier C). This result demonstrates process sensitivity, but it cannot be transferred numerically to V–Na–S deposits or converted into a valve seat service interval.

For valve seats, environmental access and mechanical damage should be qualified together. A coating that forms a protective scale during static exposure may lose that benefit when cyclic impact, microslip, or thermal strain repeatedly ruptures the scale and exposes fresh material. Conversely, a moderately hard layer with a coherent, adherent oxide can outperform a harder but defect-connected layer if the latter provides rapid paths for molten salts or hot gas. Qualification should therefore distinguish mass gain, corrosion-product mass, true metallic recession, penetration depth, spallation, and crack density rather than reporting a single “corrosion rate” that mixes different endpoints [51,56,58,59,60].

A practical environmental test sequence should also document deposit composition before and after exposure, cross-sectional chemistry and phase constitution, porosity/connectivity, edge condition, and the integrity of the layer after representative thermal cycling and contact loading. Static coupon tests remain useful for ranking chemical compatibility, but a marine valve-facing decision should progress to curved specimens or valve–seat pairs in which deposit retention, impact, heat flow, and final machining condition are reproduced. This staged approach prevents a chemically resistant coating from being qualified without demonstrating that its barrier function survives the mechanical and thermal conditions that actually control access to the substrate.

### 6.3. Thermal Fatigue, Toughness, and Interface Integrity

Thermal cycle qualification should examine crack initiation, interface separation, and retained bending or impact capacity. A Ni-rich transition layer in laser-clad Ni60B reduced reported hardness from 523.6 ± 3.5 to 397.5 ± 5.7 HV0.2 while changing the examined coating from cracked to crack-free [91] (Tier C). For Stellite 12 on H13, a high-hardness 1800 W condition (590.72 HV0.3) contained cracks and bonding defects; the study identified fewer defects at an intermediate 2200 W, while hardness at 2600 W was 487.54 HV0.3 [92] (Tier C). Neither experiment measures marine durability nor establishes a transferable fracture-toughness value. They support balancing hard phases and defect tolerance rather than maximizing hardness.

Valve-oriented Stellite thermal shock work links damage accumulation to reduced bending strength and crack initiation along heterogeneous eutectic regions [93,94,95,96] (Tier C, coating tests). The reported 350–400 °C cracking threshold in one quench protocol is a thermal shock test threshold, not a maximum service temperature [96]. A 2026 laser-power study further connects strengthening mechanisms with fracture behavior [97]. These results motivate representative heat/cool rates and dwell times on a curved valve, with residual stress and interface integrity measured after final machining. Claims of improved toughness require an identified toughness or crack-growth test; absence of visible cracks is not a numerical KIC measurement.

### 6.4. Heat Transfer, Process Selection, and Repairability

**Figure 14:**
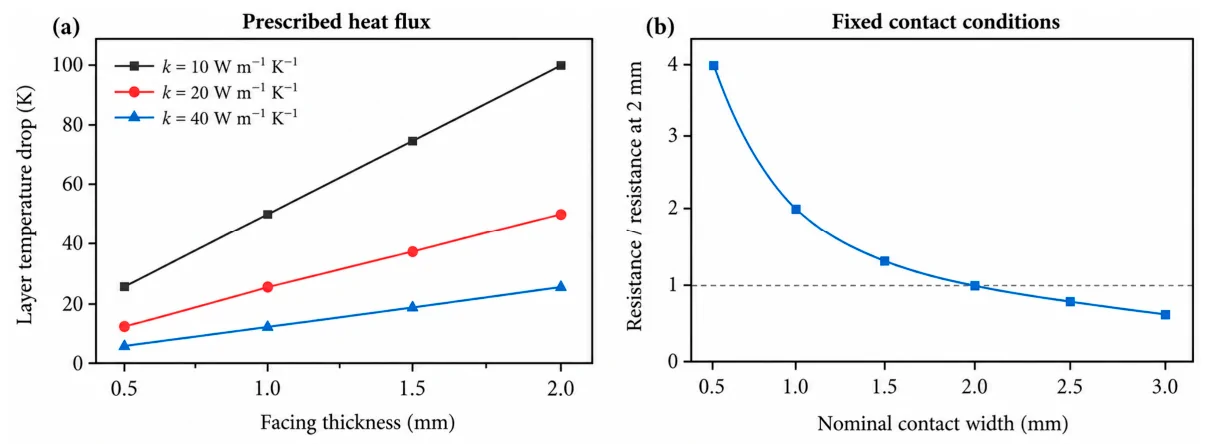
Analytical sensitivity of (**a**) the temperature drop across a facing at a prescribed heat flux of 0.50 MW m^−2^ and (**b**) normalized seat path resistance to contact width under fixed contact conditions.

A conductive seat-facing layer adds an area-specific resistance, $R_{c}^{\prime \prime }=\frac{t_{c}}{k_{c}}$. Under one-dimensional heat flux *q*^″^, the layer temperature drop is $\Delta T_{c}=\frac{q^{\prime \prime }t_{c}}{k_{c}}$. For the assumed *q*^″^ = 0.50 MW m^−2^, a 1.0 mm layer gives 50, 25, and 12.5 K at *k_c_* = 10, 20, and 40 W m^−1^ K^−1^, respectively. Across *t_c_* = 0.5–2.0 mm, the calculated range is 6.25–100 K (Figure 14). These are sensitivity calculations, not observed increases in valve-head temperature. A combustion-face barrier may reduce substrate heat input, whereas a low-conductivity seat layer may impede heat rejection. The full model must therefore include interface resistance and evolving contact.

Process choice must preserve dimensional and thermal margins. PTA provides a metallurgical bond but couples current, preheat, feed rate, dilution, and substrate heating [98,99,100,101,102,103,104] (Tier B/C). Laser routes permit localized deposition yet remain sensitive to penetration, defects, and circumferential consistency [47,105,106,107,108,109,110,111,112,113] (Tier B/C). Spraying avoids a fusion-dilution zone but requires bond and connected porosity checks; lower measured porosity is not itself proof of salt impermeability. Table 8 reports comparable process measurements with their test conditions and leaves unsupported quantities as NR.

On actual 23-8N valve specimens, PTA Stellite F was deposited to 1.1–1.4 mm, with 424.4 ± 4.3 HV0.2 reported; the associated rig used 650 °C, 10 Hz, and 5 mm lift [25] (Tier B). This is a stronger geometry match than a flat sliding coupon, but it excludes combustion chemistry and normal engine motion histories. A recent hot-wire laser/remelting study repaired 2–3 mm grooves in stainless-steel substrates using carbide-reinforced Ni-based multilayers [114] (Tier C). It demonstrates a repair route, not repeated marine valve repair life.

**Table 8:** Reported process–performance data and transfer limits for surface engineering routes.

Route, Tier, and Geometry	Process and Measured Data	Missing Data and Transfer Limit
Nitriding [25]; Tier B, valve	580 °C for 35–40 min; no fusion dilution.	Case depth, hot-hardness retention, residual stress, toughness, and porosity: NR. Time/temperature do not establish service life.
PTA Stellite F [25]; Tier B, valve	10.8 V; 110–130 A; 4–5 g min^−1^; 1.1–1.4 mm; 424.4 ± 4.3 HV0.2. Rig: 650 °C, 10 Hz, 5 mm lift.	Dilution, porosity, residual stress, and bond strength: NR. The rig omits combustion and deposits.
Laser L12/N [4]; Tier A, serviced marine valves	Finished thickness 1.2 mm. L12: 473–581 HV5 after ≈3000 h; N: 480–581 HV5 after ≈2000 h.	Hardness overlapped but damage differed. Matched-life statistics, dilution, porosity, residual stress, and bond strength: NR.
HVOF/HVAF Stellite 20 [115]; Tier C, flat coupons	610/690 HV0.3; porosity 0.41/0.32%. Dry sliding at 28 °C, 5 N, 2 Hz, 5 mm stroke, 10 min.	Thickness, connected porosity, hot hardness, bond strength, and thermal cycle life: NR. Room-temperature sliding cannot rank valve life.
Laser Ni60B with transition [91]; Tier C, coupons	1600 W; 6 mm s^−1^; 0.6 mm pass. Hardness: 523.6 ± 3.5 HV0.2 (direct, cracked) versus 397.5 ± 5.7 (transition, crack-free).	Fracture toughness, residual stress, bond strength, porosity, and repeated repair life: NR. Stress reduction was inferred, not measured.
Laser Stellite 12 [92]; Tier C, H13 coupons	1800–2600 W; 10 mm s^−1^; 40% overlap. Hardness: 590.72 HV0.3 at 1800 W and 487.54 at 2600 W; fewer defects at 2200 W.	The hardest condition contained cracks/bonding defects. Dilution, bond strength, residual stress, and hot-fatigue data: NR.

The proposed selection rule is to screen first for a sound, machinable, thermally acceptable sealing band and then for resistance to the dominant mechanism. A thick repair allowance can improve future machinability while increasing thermal resistance; a harder carbide-rich layer can reduce one wear mode while degrading crack tolerance. Matched impact–sliding, thermal cycle, and deposit tests should therefore precede a component/engine gate. This is an authors’ qualification recommendation based on mixed Tier A/B/C evidence, not a universal coating specification.

**Figure 15:**
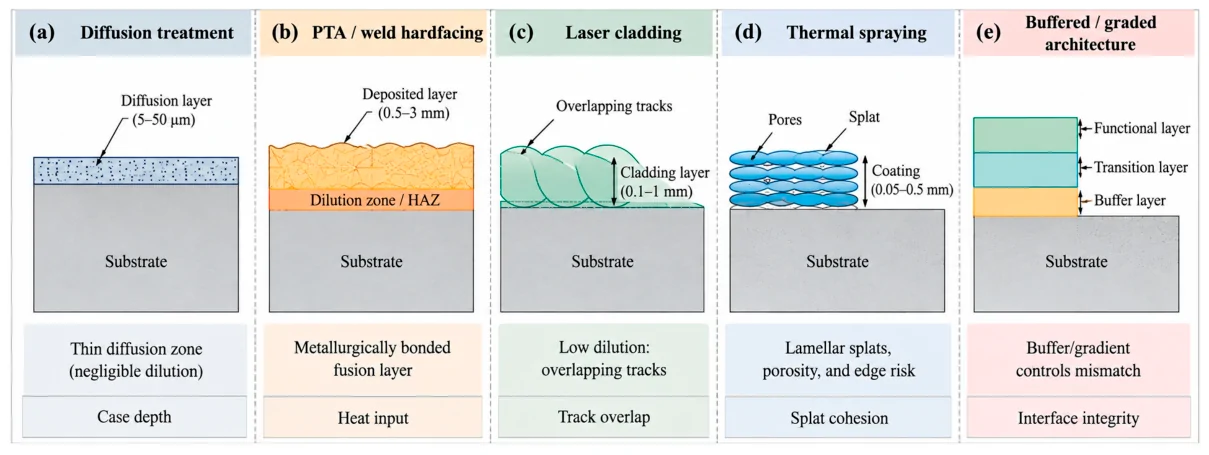
Idealized cross-sectional features of surface engineering routes: (**a**) diffusion treatment; (**b**) PTA or weld hardfacing; (**c**) laser cladding; (**d**) thermal spraying; (**e**) buffered or graded architecture.

Figure 15 compares the process-created cross-sectional features that must be included in qualification, rather than treating the nominal coating composition as the complete product definition.

### 6.5. A Repair-Qualification Workflow

Repair begins with a dimensional and metallurgical record of the serviced valve. Deep substrate cracks, unacceptable head margin, joint damage, or severe corrosion require disposition against the engine maker’s approved limits. Those limits are component-specific and are not supplied by this review. The proposed repair record includes original and current dimensions, removed layer and substrate depths, heat-treatment history, crack maps, runout, contact width, and each previous deposition cycle.

Repeated repair produces cumulative changes. If *m_j_* is the remaining structural margin after repair j, then $m_{j}=m_{0}-\sum \Delta m_{i}$ records irreversible machining loss. Deposition restores shape but not the original substrate section or microstructure. Repeated heating can overlap heat-affected zones, coarsen precipitates, alter residual stress, and move interfaces toward bending hotspots. Each cycle should re-evaluate circumferential bond integrity, heat-affected-zone microstructure and hardness, wall and head thickness, seat geometry, and thermal contact after grinding. The retained literature supports no universal maximum repair count. Table 9 should be reapplied whenever substrate history, repair depth, alloy, or process window changes.

**Figure 16:**
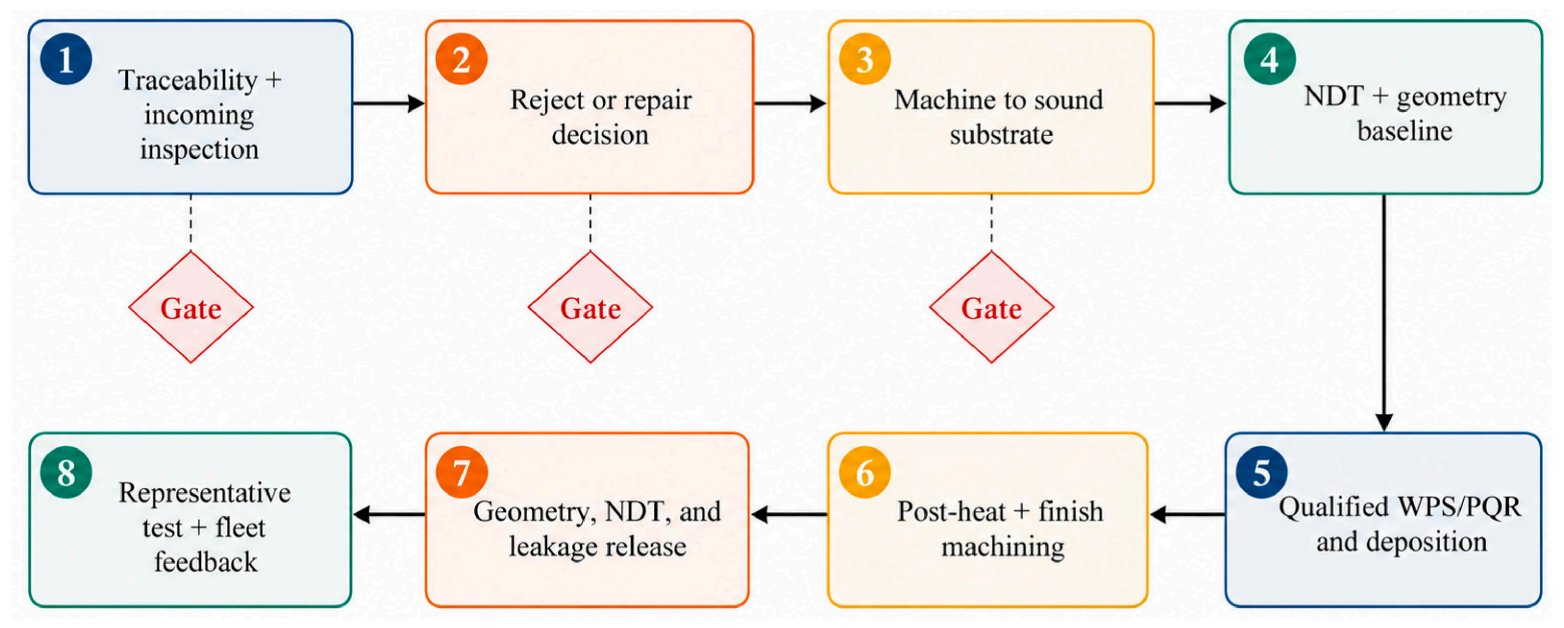
Stage-gated qualification workflow for exhaust valve remanufacturing, from service traceability to representative testing and fleet feedback. Numbers show sequence, arrows show flow, colors distinguish stages, and diamonds mark gates.

The complete stage-gated route is summarized in Figure 16; red gates prevent cosmetic restoration from being mistaken for structural and sealing qualification.

## 7. Testing, Condition Monitoring, and Life Prediction

Testing, monitoring, and prognosis form an evidence ladder. Laboratory tests identify mechanisms and process windows; component rigs establish geometric and contact effects; engine tests add combustion and installed thermal boundaries; field observations reveal operational variability and repair history; monitoring can then update the condition of an individual valve. Life prediction is defensible only when these levels are linked by measurable state variables and quantified uncertainty. This section therefore distinguishes what each validation level can establish from what remains an extrapolation.

### 7.1. Mechanism-Representative Testing

A defensible failure investigation should preserve the spatial relationship between valve, seat, deposits, cooling passages, guide, and actuator. Face recession, runout, seating width, stem straightness, guide clearance, spring load, and deposit distribution should be recorded before cleaning. Fractography, cross-sectional metallography, hardness mapping, phase and composition analysis, and oxide/deposit characterization should then be correlated with local thermal and contact histories. Operational records—fuel analyses, oil trends, cylinder pressure traces, exhaust temperature imbalance, alarms, overhaul measurements, and time since grinding or hardfacing—are part of the specimen. This interpretation is an authors’ synthesis and not an independently validated service life rule.

Accelerated testing should reproduce variables that control damage rather than merely maximize temperature or load. A representative valve seat test combines the mean face temperature and through-head gradient; start–stop and load-change transients; normal impact and pressure-induced tangential microslip; relevant gas and deposit chemistry; valve rotation; and a defensible relationship between test and service cycles. Endpoints should include leakage area or flow, recession, contact-width evolution, face distortion, crack density, volume loss, deposit penetration, oxide continuity, hardness retention, and interfacial integrity. This interpretation is an authors’ synthesis and not an independently validated service life rule.

Table 9 compares what is reproduced at coupon, complete-component, engine, and field levels. A coupon result can screen one mechanism; a valve rig can add contact geometry and repeated impact; an engine can add combustion, cooling, and transients; field exposure can test operational variability and maintenance. None automatically replaces the others. Agreement in morphology, contact evolution, temperature, and leakage is more persuasive than equal nominal temperature or one acceleration factor. Replication, uncertainty, healthy controls, and censored components should be reported at every applicable level (authors’ validation recommendation).

### 7.2. Leakage and Valve-Event Monitoring

Marine experiments with deliberately introduced valve leakage demonstrate changes in combustion and emission indicators [70] (Tier A, engine-level fault detection). Such signals can flag a sealing anomaly, but they do not uniquely identify corrosion, a crack, or recession depth. Their baseline must account for load, injection, sensor drift, and other cylinder faults.

Acoustic-emission, vibration, and signal-classification studies demonstrate leakage or clearance diagnosis in particular engines or test arrangements [116,117,118,119,120,121,122,123] (Tier A/B). Their established task is fault detection or classification under the tested conditions. Accuracy from a controlled fault set should not be represented as prospective marine fleet sensitivity or converted into remaining fatigue life. Independent engine validation and checks for changes in sensor mounting and duty are required before transfer.

A practical system should fuse crank-angle-referenced AE or vibration with cylinder pressure, exhaust temperature, speed, load, fuel command, and maintenance history. Event windows around opening and seating retain more physical meaning than whole-cycle statistics. Features should be normalized against an engine-specific healthy baseline, and an alert should require agreement among independent symptoms—for example, a progressive seating-event change, persistent exhaust-temperature deviation, and pressure evidence of leakage. Sensor coupling, propagation-path changes after maintenance, injector noise, and missing operating coverage belong in the uncertainty model. This interpretation is an authors’ synthesis and not an independently validated service life rule.

### 7.3. Physics-Informed Prognosis and Digital Twins

**Figure 17:**
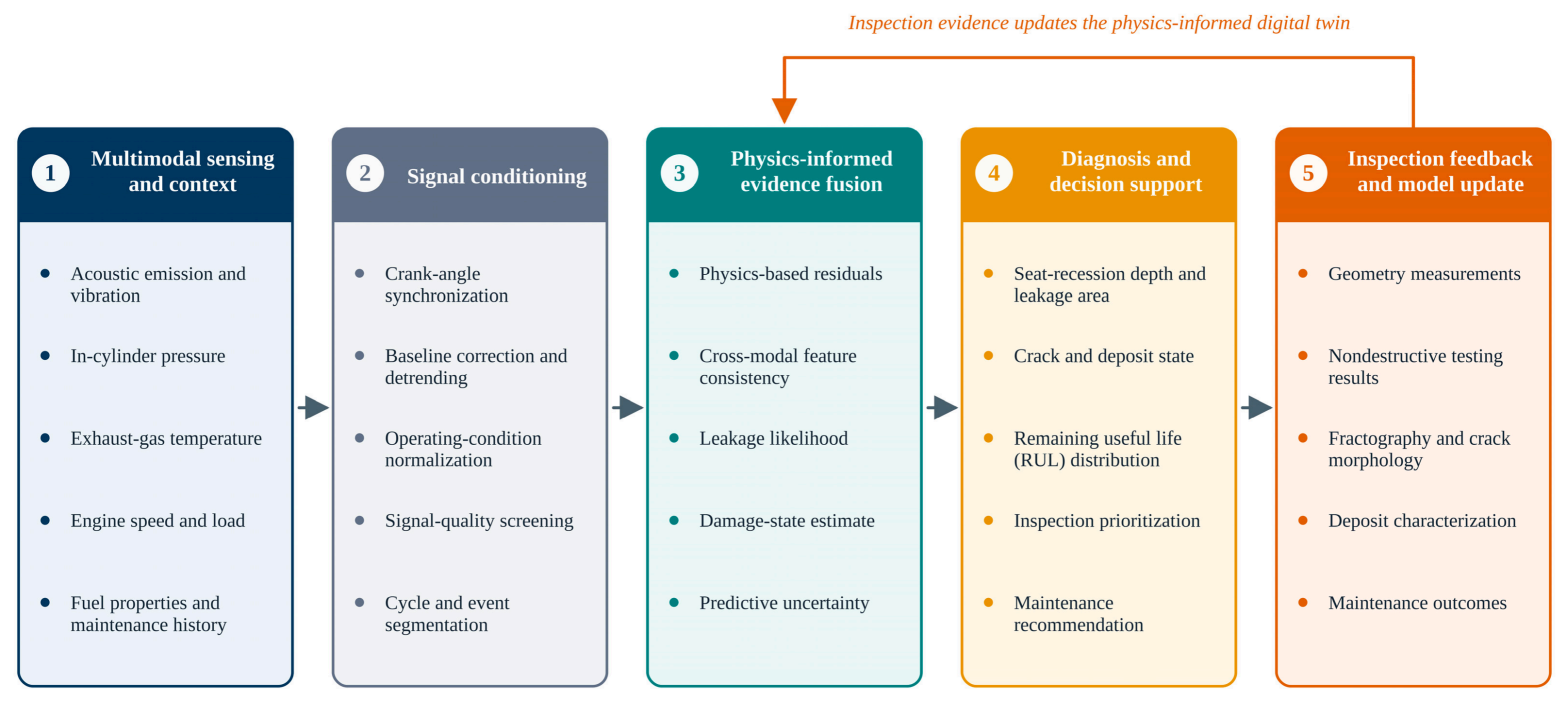
Proposed physics-informed monitoring and prognosis architecture linking multimodal sensing, event-resolved features, engine-specific baselines, latent damage states, probabilistic remaining life, and inspection feedback. Numbers show stages; gray arrows show workflow; orange shows feedback; colors distinguish stages.

Physics-informed valve prognosis remains a research objective rather than a validated service tool. Candidate latent state variables include contact width, leakage area, recession, crack size, deposit/oxide condition, local temperature, and retained working-layer thickness. These states could in principle be updated using cylinder pressure, exhaust temperature, crank-angle-resolved acoustic or vibration features, and scheduled inspection measurements. However, an actionable remaining-life estimate also requires a calibrated damage law, a validated observation model linking sensor features to physical state, uncertainty propagation, censoring information, and prospective failure data; these requirements are not supplied by the conceptual architecture in Figure 17.

Marine engine digital twins have been reported for performance prediction, diagnostic analysis, health assessment, and model trustworthiness [124,125,126,127,128] (Tier A, engine-level modeling), and a 2026 transfer-learning study further demonstrates data-driven updating of marine engine prediction models [129] (Tier A). For valve degradation, a useful digital twin would require at least three connected levels: a boundary-condition estimator that reconstructs thermal and operating exposure; a damage-state estimator that assimilates inspection and event-resolved sensing; and a decision layer that reports risk or remaining-useful-life distributions rather than a single deterministic value. Calibration on healthy operation is not equivalent to validation of degradation progression. Independent engine testing, explicit model-discrepancy terms, sensor-fault handling, and the ability to abstain when operating conditions fall outside the training or calibration envelope are therefore essential before valve-specific prognosis can support maintenance or repair release.

The validation target should also match the intended decision. A model intended only to prioritize inspection can be evaluated by ranking accuracy and missed-damage risk, whereas a remaining-life model requires time-to-event calibration, uncertainty coverage, and prospective validation on valves that were not used for model development. Repair history is particularly important because machining, deposition, and repeated heat input change the initial state from which future damage accumulates. For near-term deployment, an uncertainty-aware risk index tied to inspection thresholds is therefore more defensible than a precise autonomous life prediction.

Figure 17 presents the proposed architecture for future valve-specific prognosis; the latent-state and remaining-life links require prospective validation.

## 8. Integrated Mechanism–Material–Process–Performance Framework

The proposed workflow starts with the valve’s fuel/pilot-fuel and lubricant exposure, duty, peak pressure, contact geometry, heat rejection, and overhaul requirements. Table 10 applies it to a worked hardfacing selection using the published marine L12/N comparison [4]. This is an evidence-based retrospective illustration, not a new comparative experiment or approval of a commercial coating (Tier A).

The matrix applies four gates: adequate environmental resistance; retained sealing/wear geometry; thermal and crack tolerance; and manufacturability with repair allowance. Evidence can support, oppose, or leave a gate unresolved. A weighted score is avoided because the available reports do not provide comparable lifetime distributions or defensible weights. An unresolved critical gate remains a request for qualification rather than being silently counted as a pass.

In the worked example, the N-facing service damage is adverse evidence for the observed combination of facing and duty. L12 is the better-supported candidate for further qualification, because less damage was observed over a longer reported exposure; its observation is censored and does not define mean life. Coupon alternatives with higher hardness have lower application proximity and cannot displace this evidence without matched component and engine tests. Final selection still requires the actual fuel/deposit specification, thermal/contact measurements, dimensional acceptance limits, and inspection results.

**Figure 18:**
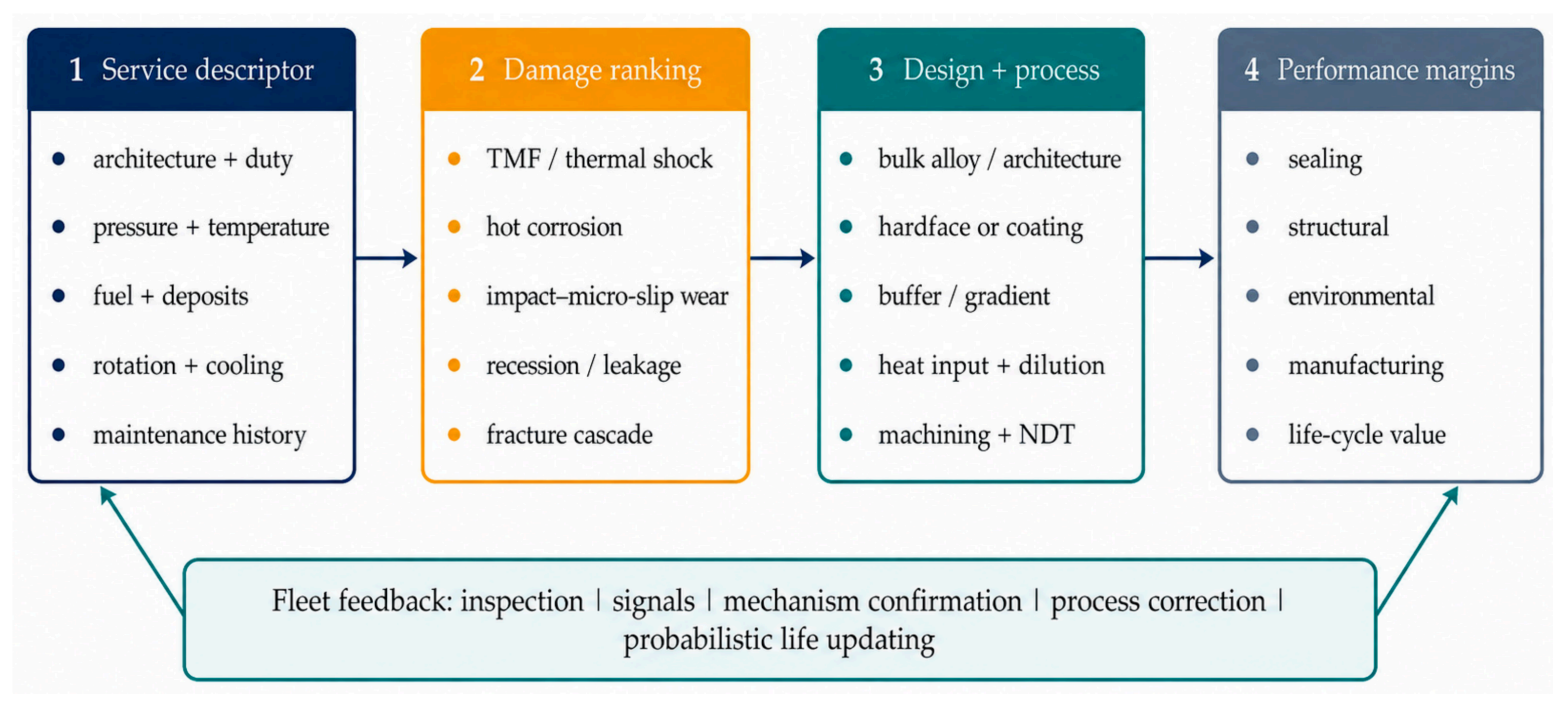
Mechanism–material–process–performance framework for valve design, remanufacturing qualification, and evidence-based fleet feedback.

Figure 18 summarizes the proposed decision workflow, with the worked evidence gates in Table 10 and a future fleet feedback loop.

The practical output of the framework is thus a traceable candidate and a list of unresolved gates. Tier A marine-specific evidence informs context-specific decisions, but its experimental or modeling limitations still apply; Tier B supports mechanistic transfer, and Tier C identifies material/process hypotheses. A service interval or repair release requires application-specific qualification beyond this review.

## 9. Research Gaps and Outlook

The most important research needs are not simply additional material property measurements, but the closure of the transfer gaps identified throughout this review: from coupon to valve geometry, from isolated to coupled mechanisms, from new to repeatedly repaired components, and from fault detection to validated prognosis. The following priorities are organized around the evidence needed to make material selection, remanufacturing, monitoring, and maintenance decisions traceable and transferable to medium-speed marine service.

### 9.1. Valve-Specific Marine Datasets

The foremost gap is the scarcity of shareable, valve-specific datasets for medium-speed marine engines. Existing studies rarely link material heat, manufacturing or repair history, fuel and lubricant chemistry, duty, sequential inspection, onboard signals, and final destructive examination. Multivessel datasets should include healthy, failed, repaired, and right-censored valves. A common schema should record valve seat geometry and pairing, material and process identity, repair count, deposit chemistry, recession, leakage, crack metrics, and synchronized operating data. Definitions, units, uncertainty, and missing-data flags should be standardized to support unbiased life models and the proposed evidence hierarchy. This is an authors’ synthesis, not a validated service life rule.

### 9.2. Coupled Test Protocols and Mechanism Transitions

Thermal fatigue, impact–sliding wear, oxidation, hot corrosion, creep, and leakage erosion are usually studied separately, despite their service coupling. Future rigs should reproduce transitions from protective film formation to rupture, recession, leakage, or crack initiation. Acceleration is defensible only when it preserves contact pressure, tangential displacement, thermal gradient amplitude and phase, deposit chemistry, leakage state, damage morphology, and state trajectory. Interrupted examinations and interlaboratory round-robin tests on a shared valve seat geometry could distinguish material effects from fixture-specific behavior. This is an authors’ synthesis, not a validated service life rule.

### 9.3. Fuel Transition and Deposit Chemistry

Recent studies provide distinct evidence on fuel transitions rather than a direct ranking of valve life. Methanol–diesel CFD addresses combustion and emissions [48], Nimonic 80A coupons in ammonia show temperature-dependent nitridation [49], and cast-iron liner tests address a different corrosion regime [50]. Future comparisons should measure pilot-fuel fraction, lubricant carry-over, water activity, unburned species, oxygen potential, deposits, and near-wall temperature across load changes. The resulting films must then be tested under representative seating impact and microslip. The bounded 2024–2026 update improves recency but is not exhaustive.

### 9.4. Scalable Surface Engineering and Repeated Repair

Multilayer and graded concepts require full-circumference qualification on previously serviced valves, not only successful local cross-sections on virgin coupons. PTA, laser, spray, and graded routes should map layer thickness, dilution, heat-affected zone width, connected porosity, residual stress, microstructure, interface position, and final seat geometry. Process windows must include preheat, deposition energy, material delivery, remelting or interpass history, and machining allowance [91,92,98,99,100,101,102,103,104,105,106,107,108,109,110,111,112,113,114]. Repeated repair studies should use thermally aged or previously repaired substrates and track cumulative material removal and heat input. This is an authors’ synthesis, not a validated service life rule.

### 9.5. Multi-Fidelity Prognosis with Explicit Uncertainty

Valve prognosis should separate model verification, parameter calibration, state estimation, and decision validation. High-fidelity thermal, contact, and corrosion models can define sensitivities, while reduced-order models update physically meaningful states from onboard data and inspections. Uncertainty must cover sensor error, model-form discrepancy, repair history, variable fuel or deposit exposure, and sparse failure data. Existing digital-twin and transfer-learning studies show engine-level updating capabilities [124,125,126,127,128,129], but valve prognosis still requires prospective independent engine validation. Near-term tools should prioritize inspections and report false-negative risk, calibrated uncertainty, and out-of-domain operation. Autonomous remaining-life decisions should await valve-specific validation.

## 10. Conclusions

This review synthesizes evidence showing that medium-speed marine exhaust valve durability depends on coupled thermal, mechanical, tribological, chemical, and heat transfer processes. Direct medium-speed marine component evidence is distinguished from marine studies with different speed classes or endpoints, Tier B heavy-duty diesel/gas-engine/valve-test analogs, and Tier C generic materials/coating evidence. Because medium-speed marine component datasets remain scarce, some conclusions necessarily rely on Tier B/C evidence for mechanism identification or screening, not service life transfer; several qualification and prognosis recommendations therefore remain conceptual rather than quantitatively validated life relationships. The main conclusions are:

1. Valve degradation arises from interacting thermal, mechanical, tribological, and environmental processes that drive face loss, fatigue cracking, corrosion, leakage, and sealing deterioration.
2. Reported temperature, contact, and leakage values are condition-specific. Their experimental or modeling basis must be retained, and they should not be treated as universal limits or life predictors.
3. Bulk material selection should prioritize retained hot strength, fatigue and creep resistance, thermal properties, corrosion resistance, and manufacturing history rather than room-temperature hardness or strength alone.
4. Surface performance is governed by the coating–interface–substrate–seat system. Thickness, conductivity, dilution, porosity, interface integrity, and repair history are as important as hardness.
5. The mechanism–material–process–performance framework provides a traceable basis for qualification but is not a validated service life model. Priority needs are representative marine tests, coupled thermal–contact–leakage measurements, repeated repair studies, and validated monitoring.

Overall, material selection and repair release should shift from property-based ranking to mechanism- and service-state-based qualification.

## Figures and Tables

**Table 1:** Evidence tiers used in the narrative synthesis.

Tier	Evidence Proximity	Permitted Inference
A	Marine field, engine, or explicitly identified marine model	Highest application proximity; speed class, duty, method, and endpoint still limit transfer. Models and diagnostics do not validate life.
B	Valve seat tests, non-marine engines, or matched-environment analogs	Supports mechanisms and preliminary ranges; differences in fuel, cooling, geometry, speed, and duty preclude direct marine life claims.
C	Generic coupons, materials/process studies, reviews, standards, or bulletins	Supports screening, context, and assumptions; does not establish component performance.

Each cited source is assigned once in Supplementary Table S2. Tier A covers explicitly identified marine field, engine, simulation, or monitoring studies; unreported speed still limits transfer to medium-speed service. Tier B covers non-marine valve tests, heavy-duty or gas engine analogs, and engine-matched corrosion simulations. Tier C includes generic coupons, reviews, standards, methods, and manufacturer data. The register contains 17 Tier A, 68 Tier B, and 44 Tier C sources, not independent experiments. Major figures and quantitative tables state both tier and study mode. Authors’ syntheses, calculations, and proposed criteria retain that label and are not promoted by citation to a marine case.

**Table 2:** Basis and transfer limits of reported temperature examples (Tier A/B).

Source and Evidence	Engine or Boundary Conditions	Reported Temperature and Limitation
Yang et al. [1]; Tier A, marine FEA with indirect hardness comparison	Ni80A; 500 rpm; 320 mm bore; 1324 kW; peak pressure 9.6 MPa. Seat boundary: 653.1 K and 5000 W m^−2^ K^−1^; contact during 60% of the cycle.	Peak ≈ 700 °C from FEA; hardness provided an indirect comparison. Furnace calibration: 10 h at 320–760 °C. No numerical uncertainty interval.
Deng et al. [2]; Tier A, marine FEA	Closed valve; peak pressure 20 MPa. Gas-side zones: 1100 °C/523 and 800 °C/700 W m^−2^ K^−1^; seat: 300 °C/3605 W m^−2^ K^−1^. Speed/load: NR.	Peak 712 versus 693 °C. No direct temperature measurement or uncertainty interval; geometry and material changes are confounded.
Lai et al. [25]; Tier B, gasoline-engine dynamometer	Solid and hollow-stem sodium-filled valves; 2 h at the reported full-speed condition. Numerical speed, load, torque, and cooling boundaries: NR.	Indirect hardness thermometry gave ≈ 745 versus 590 °C. No propagated uncertainty; the separate 16.1% mass reduction is not a marine validation.

**Table 3:** Fuel and lubricant pathways to deposits and valve damage.

Fuel and Carry-Over	Exposure to Verify	Mechanism and Evidence Limit
HFO; lubricant and sea salt	Measure V, Na, S, Ca, P, and Zn; identify sulfate/vanadate phases and molten fraction.	Scale fluxing and internal sulfidation, followed by impact or leakage removal [8,9,40,41] (Tier B). HFO does not define a fixed chemistry.
Distillate; lubricating oil	Measure lubricant residues, remaining sulfur, and contaminants.	Tribofilm evolution and oxidation remain relevant [31,42] (Tier B). Regulatory sulfur limits [43,44] (Tier C) do not predict metal loss.
LNG dual fuel; pilot fuel and oil	Measure pilot/oil ash, water exposure, and contact tribofilm; verify any V-rich deposit.	Gas-engine oxide, tribofilm, and seating evidence [45,46,47] (Tier B/C) differs from residual fuel marine duty.
Methanol dual fuel; pilot fuel and oil	Measure water, unburned oxygenates, condensate chemistry, and pilot/oil deposits.	High-speed marine CFD [48] (Tier A) does not measure hot-valve loss; condensate corrosion and hot-seat oxidation require separate tests.
Ammonia; pilot fuel and oil	Measure unburned NH_3_, water/oxygen activity, nitrides, and carry-over deposits.	Nimonic 80A nitridation [49] and cast-iron liner corrosion [50] (Tier C) concern different exposures and cannot quantify hot-valve loss.

**Table 4:** Valve zones, dominant loads, damage signatures, and diagnostic evidence.

Zone	Primary Load	Typical Damage	Confirmation
Combustion face	Gas heat flux, pressure, oxidation, deposits	Scale, pits, guttering, dish distortion	Deposit/oxide chemistry, thickness map, profile, flatness
Seat-contact band	Impact, pressure-driven microslip, intermittent cooling	Recession, polishing, transfer, pits, radial cracks	Contact pattern, runout, wear profile, SEM/EDS, leakage test
Head–neck fillet	Bending, thermal gradient, stress concentration	Circumferential or radial TMF cracks	Fractography, thermal–stress model, hardness/microstructure map
Stem–guide pair	Sliding, side thrust, heat-transfer, lubrication	Scuffing, ovality, bending, seizure	Clearance, straightness, surface chemistry, lubricant/deposit analysis
Tip/keeper grooves	Contact, spring load, assembly, misalignment	Peening, fretting, groove cracking	Dimensions and dye-penetrant or magnetic-particle testing

**Table 5:** Environmental exposure data and endpoint-specific limitations.

Material and Source	Exposure	Outcome and Transfer Limit
Nimonic 80A [8]; Tier B, diesel environment simulator	700 °C without ash versus 750 °C with ash aerosol and SO_2_; duration and full ash recipe unavailable.	Damage zone: 0 versus 135 µm, including 30 µm internal sulfidation. Temperature and chemistry both changed; no rate can be calculated.
SEH3 valve steel [41]; Tier B, exhaust-fed furnace	Fuel sulfur 0.61 wt%; source engine 2000 rpm/40 PS; specimens 9 × 100 mm; 900–1100 °C; V 1–100 ppm.	At 1000 °C, descaled loss $W=13.5\sqrt{t}$ mg cm^−2^ (t in h); 2 h loss rose ≈50-fold from 900 to 1100 °C. No isolated Na series.
Nimonic 80A [49]; Tier C, static coupons	80 vol% NH_3_/Ar; 50 sccm; 500 or 800 °C; 144 h; 20 × 10 × 5 mm; *n* = 3 per condition.	Nitridation penetration ≈ 17 and 37 µm. CrN formation and hardness change are not metal loss; mass gain is not a recession rate.
Cr_3_C_2_–NiCr HVOF [51]; Tier C, coupons	45% NaCl–55% KCl; 600 °C; 100 h; milled versus unmilled powder.	Product mass: 3.40 versus 10.07 mg cm^−2^. Salt chemistry differs from V–Na–S; product mass is not recession. Room-temperature erosion is not hot-valve wear.

**Table 6:** Evidence supporting and limiting the proposed leakage–damage feedback.

Link	Available Evidence	Unresolved Requirement
Contact geometry → heat rejection	Marine thermal models [1,2] (Tier A); valve heat/contact studies [20,21,22,23,24] (Tier B).	Measure pressure, deposits, and intact contact fraction; width alone does not determine conductance.
Chemistry/temperature → surface damage	Engine-matched corrosion simulation [8,41] (Tier B); post-service marine faces [4] (Tier A).	Separate tests support mechanisms, not one universal sequence or rate.
Leakage → combustion/exhaust response	Imposed-leak marine engine test [70] (Tier A).	Local jet heat deposition and valve-metal temperature remain unmeasured.
Damage → recession/leak growth	Valve wear/contact studies [24,36,37,66] (Tier B); damaged marine faces [4] (Tier A).	Calibrate coupled corrosion–impact coefficients and evolving gap geometry.
Leakage/overheating → fatigue/fracture	Fatigue and terminal fracture in a marine casualty [3] (Tier A).	The preceding leakage/temperature history is unknown; other initiation routes remain possible.

**Table 7:** Partial, condition-specific comparison of reported bulk valve material properties; property coverage is incomplete, and the table should not be interpreted as a comprehensive material ranking. UTS, ultimate tensile strength; E, elastic modulus; CTE, coefficient of thermal expansion; NR, not reported.

Material and Condition	Mechanical Data	Thermal/Environmental Limits
42Cr9Si2 [53]; Tier B; quenched 1030 °C/0.5 h in oil; tempered 650 °C/1.5 h in air	400 °C: UTS/yield 959 ± 13/792 ± 18 MPa; E 196 ± 14 GPa. 650 °C: 512 ± 12/454 ± 15 MPa; E 137 ± 9 GPa. 10^7^-cycle strength: 569 to 300 MPa.	Room-temperature hardness 37 HRC. Creep, conductivity, CTE, fracture toughness, and deposit-corrosion rate: NR. Isothermal fatigue is not TMF.
23-8N [25]; Tier B; source valve-steel condition	UTS/yield: 1002/605 MPa at 25 °C and 579/310 MPa at 650 °C. E: 183 to 149 GPa. Creep and fracture toughness: NR.	Conductivity and CTE at matching temperatures: NR. Facing hardness must not be used as substrate hot hardness.
NCF3015 [32,68,74]; Tier B; Fe–Ni valve alloy	Valve tribology and forging evidence; comparable tensile, creep, and fatigue data: NR.	Oxide/tribofilm and microstructure depend on exposure and processing. Conductivity, CTE, hot hardness, and fracture toughness: NR.
Nimonic 80A [75]; Tier C; forged-bar bulletin	E ≈ 175 GPa at 700 °C and 165 GPa at 800 °C. Matched tensile, creep, and fatigue data were not extracted.	Conductivity: 11.2 W m^−1^ K^−1^ at 20 °C and 22.3 at 700 °C. Mean CTE, 20–700 °C: 15.5 × 10^−6^ K^−1^. Sulfidation/nitridation: [8,49].
Inconel 751 [52]; Tier C; directly aged 732 °C/2 h	UTS/yield: 1100/889 MPa at 649 °C and 554/526 MPa at 816 °C. 100 h rupture stress: 476/193 MPa; extrapolated 1000 h: 276/96.5 MPa.	Hardness: 352 BHN at room temperature, 291 at 649 °C, 173 at 816 °C. Conductivity 12.0 W m^−1^ K^−1^. Bulletin values are not guaranteed allowables.

**Table 9:** Validation levels and the decisions they can support.

Validation Level	Reproduced Variables and Endpoints	Supported Decision and Remaining Limits
Coupon [49,91,92,115]; Tier C	Composition, local microstructure, selected temperature/atmosphere, hardness, porosity, sliding, or cracking.	Screens mechanisms. Usually omits curvature, circumference, impact, engine heat flux, deposits, and repair history; cannot release a valve.
Complete valve seat rig [25,53]; Tier B	Actual geometry, impact, contact evolution, and heated cycling; examples include 650 °C, 10 Hz, 5 mm lift.	Gates dimensional stability and wear/crack morphology. Does not reproduce full combustion chemistry or fleet duty.
Engine test [25] Tier B; [70] Tier A	Combustion, installed cooling, valve motion, defined duty, temperature, or imposed-leak response.	Gates thermal and sealing performance under the tested schedule; limited engines and duration constrain transfer.
Field/service examination [3,4]; Tier A	Real fuel/oil, duty variability, maintenance, morphology, and reported observation time.	Establishes application relevance. Fleet reliability requires healthy/censored controls and prospective follow-up.

**Table 10:** Worked evidence-gate comparison of N and L12 hardfacings for a marine exhaust valve.

Requirement	N Versus L12 Evidence [4] (Tier A)	Decision and Unresolved Qualification
Retain sealing geometry	N suffered severe damage at ≈2000 h; L12 showed less damage at ≈3000 h, with censoring.	Advance L12 for this application. Do not infer a universal 1.5-fold life or fleet failure probability.
Hot hardness and crack tolerance	Near-surface hardness overlapped; field data did not isolate toughness, defects, or chemistry.	Hardness does not discriminate. Add matched hot impact–sliding, thermal cycle, and bond tests.
Environmental resistance	Marine provenance is relevant, but no controlled chemistry-matched comparison is available.	Measure actual fuel/deposit chemistry and reproduce it comparatively; gate unresolved.
Heat rejection and contact	Finished facing thickness was 1.2 mm; conductivity and evolving contact were not reported.	Measure conductivity, contact width, and runout; assess installed thermal margins. Figure 14 gives sensitivity only.
Manufacture and repeated repair	Service records do not validate multiple repairs.	Record removal depth, heat-affected zone, interface, and remaining dimensions; requalify after material changes.
Higher-hardness coupon option	H13/Stellite 12 [92] (Tier C) provides process/hardness data without a marine sealing endpoint.	Retain for screening until component, engine, and field gates in Table 9 are satisfied.

## Data Availability

No new data were created or analyzed in this study. Data sharing is not applicable to this article.

## Supplementary Materials

The following supporting information can be downloaded at https://www.mdpi.com/article/10.3390/ma19194166/s1: Table S1: Search queries and retrieved records; Table S2: Evidence-tier assignment for cited sources; Table S3: Quantitative extraction notes.
